# The Effect of Ethanolic Extract of Brazilian Green Propolis and Artepillin C on Cytokine Secretion by Stage IV Glioma Cells Under Hypoxic and Normoxic Conditions

**DOI:** 10.3390/ph18030389

**Published:** 2025-03-09

**Authors:** Małgorzata Kłósek, Anna Kurek-Górecka, Radosław Balwierz, Grażyna Pietsz, Zenon P. Czuba

**Affiliations:** 1 Department of Microbiology and Immunology, Faculty of Medical Sciences, Medical University of Silesia in Katowice, Jordana 19, 41-808 Zabrze, Poland; akurekgorecka@sum.edu.pl (A.K.-G.); gpietsz@sum.edu.pl (G.P.); zczuba@sum.edu.pl (Z.P.C.); 2 Institute of Chemistry, University of Opole, Oleska 48, 45-052 Opole, Poland; radoslaw.balwierz@uni.opole.pl

**Keywords:** propolis, artepillin C, cytokine, normoxia, hypoxia, glioma cells

## Abstract

**Background:** The majority of gliomas are astrocytic in nature. Gliomas have the lowest survival rate among all tumors of the central nervous system (CNS), characterized by high aggressiveness and poor response to treatment. The tumor microenvironment is a source of cytokines such as IL-6, IFN-γ, VEGF, and PDGF-BB, secreted mainly by tumor and immune cells. These cytokines play a significant role in angiogenesis, invasion, and metastasis formation. In vitro and in vivo studies have shown that Brazilian green propolis, derived from *Baccharis dracunculifolia* DC and rich in artepillin C, exhibits anti-inflammatory, antimicrobial, chemopreventive, and anticancer activities. Additionally, it can penetrate the blood–brain barrier, demonstrating neuroprotective effects. The aim of the present study was to determine the concentration of selected cytokines produced by astrocytes of the CCF-STTG1 cell line, isolated from the brain of a patient with stage IV glioma (astrocytoma). **Methods:** The cytotoxicity of the EEP-B was evaluated using the MTT assay. Astrocytes were stimulated with LPS at a final concentration of 200 ng/mL and/or IFN-α at 100 U/mL, followed by incubation with EEP-B (25–50 µg/mL) and artepillin C (25–50 µg/mL) under 2-h hypoxia and normoxia conditions. Cytokine concentrations were measured using the xMAP Luminex Multiplex Immunoassay and the Multiplex Bead-Based Cytokine kit. **Results:** The absence of cytotoxic effects of EEP-B and artepillin C on human astrocytes of the CCF-STTG1 lineage was demonstrated. Stimulation with LPS, IFN-α, and their combination (LPS + IFN-α) significantly increased the secretion of the tested cytokines compared to the control cell line. The most pronounced and statistically significant reduction in cytokine levels, particularly IL-6 and VEGF, was observed following EEP-B treatment at both tested concentrations under both hypoxic and normoxic conditions. **Conclusions:** Brazilian green propolis may serve as a potential immunomodulator in combination therapies for gliomas of varying malignancy grades.

## 1. Introduction

The largest group of malignant tumors of the central nervous system are gliomas. Based on the tumor’s growth rate and histological characteristics, the World Health Organization (WHO) classifies gliomas into four grades of malignancy, ranging from the least aggressive (grade I) to the most aggressive (grade IV). The majority of gliomas are astrocytic in nature. These gliomas have the lowest survival rate among all central nervous system (CNS) tumors [1,2,3]. The standard treatment for gliomas includes a combination of surgery, radiotherapy, and chemotherapy. For malignant gliomas, chemotherapy primarily involves the use of temozolomide [4]. Additionally, high levels of hypoxia in tumor tissue play a significant role in glioma progression and resistance to treatment.

Hypoxia is a condition characterized by low oxygen concentration in tissues and is commonly observed in most solid tumors, including those of the colon, breast, liver, pancreas, and central nervous system (CNS) [5,6,7,8,9]. In most cells, hypoxia occurs when the oxygen concentration drops below 2%. This condition activates the heterodimeric transcription factor HIF-1 (hypoxia-inducible factor 1), whose elevated expression in cancer cells contributes to cancer progression [10,11,12]. Hypoxia drives these effects by altering cell metabolism, regulating angiogenesis, impairing immunosurveillance, and accelerating metastasis development.

The tumor microenvironment is characterized by a diverse array of cells and involves numerous interactions between tumor cells, the surrounding stroma, immune cells, and cytokines. This microenvironment plays a critical role in promoting tumor growth [13,14]. A significant group of mediators in this process are cytokines, a family of protein molecules involved in all stages of carcinogenesis. They act as essential growth and survival factors for tumor development. Furthermore, cytokines induce changes in the tumor microenvironment and play a key role in invasion and metastasis. The tumor microenvironment serves as a source of, inter alia, interleukin 6 (IL-6), interferon gamma (IFN-γ), vascular endothelial growth factor (VEGF), or platelet-derived growth factor (PDGF-BB).

In the CNS, astrocytes are a major inducible source of interleukin-6. IL-6 plays a crucial role in the progression of brain tumors by promoting angiogenesis, stimulating cell proliferation, enhancing resistance to apoptosis, and supporting cancer development. Overexpression of IL-6 contributes to neuropathology associated with CNS inflammation [15,16]. VEGF is an angiogenic factor that promotes the formation of new blood vessels in the tumor microenvironment and increases the permeability of the blood–brain barrier (BBB) by downregulating tight junction proteins. The PDGF family includes PDGF-AA, AB, BB, CC, and DD. Overexpression of PDGF, particularly PDGFB and PDGFA, plays an important role in the genesis and progression of glioblastoma [17]. IFN-γ, a member of the type II IFN family, is an important cytokine present in the tumor microenvironment. It plays a critical role in regulating immune response, chemotactic signaling, antigen presentation, and inflammation, thereby influencing metastasis [18]. IL-5 produced by astrocytes may mediate interactions between brain cells and immune cells [19].

Propolis is a natural, sticky substance produced by bees, consisting of a mixture of plant resins, beeswax, pollen, bees’ glandular secretions, and mechanical admixtures [20]. The chemical composition of propolis depends on the local flora at the collection site. Brazilian green propolis, derived from *Baccharis dracunculifolia*, is rich in artepillin C. Numerous in vitro and in vivo studies have demonstrated that it possesses anti-inflammatory, antibacterial, chemopreventive, anticancer, or neuroprotective effects [21,22].

Astrocytes are the most abundant glial cells in the brain and play a key role in regulating the inflammatory response in the central nervous system. The aim of our study was to determine the concentration of selected cytokines produced by astrocytes under an ethanolic extract of Brazilian green propolis and artepillin C stimulated with LPS and/or IFN-α during 2-h hypoxia, compared to normoxic conditions. The examined cells were isolated from the brain of a patient with grade IV astrocytoma. In this study, we sought to explore the immunomodulatory effect of propolis against cancer cells under hypoxic conditions. For the first time, we compared the cytokine profile produced by astrocytes in grade IV malignancy under hypoxia and normoxia when exposed to Brazilian green propolis and artepillin C with LPS and/or IFN-α. Our research hypothesis is that the immunomodulatory action of Brazilian green propolis and its active ingredient, artepillin C, could be used in cancer immunotherapy.

## 2. Results

### 2.1. The Viability of CCF-STTG1 Astrocytes After Stimulation with an Ethanolic Extract of Brazilian Green Propolis (EEP-B) in Normoxia and Hypoxia Conditions

**Figure 1:**
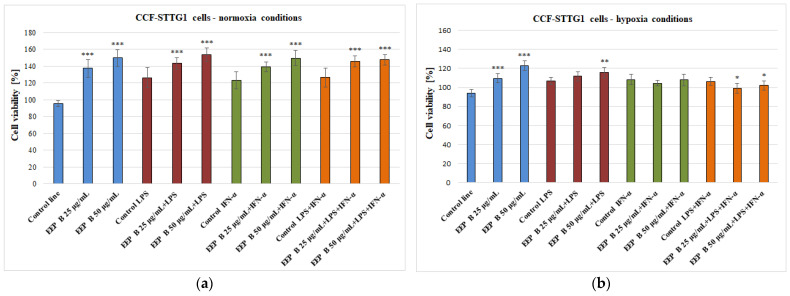
Cell viability [%] measured by MTT—CCF-STTG1 cells in combination with ethanolic extract of Brazilian green propolis (EEP B) with LPS, IFN-α, or LPS + IFN-α in normoxia (**a**) and hypoxia (**b**) conditions. The values represent the means ± SD of three independent assays: * *p* < 0.05, ** *p* < 0.01, and *** *p* < 0.001 compared to adequate control.

The CCF-STTG1 cells were incubated with an ethanolic extract of Brazilian green propolis at final concentrations of 25–50 μg/mL for 24 h. EEP B increases the viability of cells from 95.73 ± 3.70% to 149.92 ± 9.66% incubated under normoxia (Figure 1a) and from 94.08 ± 3.93% to 122.89 ± 5.35% incubated under hypoxia (Figure 1b) conditions. In normoxia conditions, the ethanolic extract of Brazilian green propolis with LPS, IFN-α, or LPS + IFN-α resulted in a significant increase in the viability of the examined cells at both concentrations (Figure 1a). In hypoxia, EEP B at both tested concentrations with LPS + IFN-α caused a decrease in cell viability from 106.37 ± 4.05% to 99.32 ± 5.18% and 102.05 ± 4.82% accordingly (Figure 1b).

### 2.2. The Viability of CCF-STTG1 Astrocytes After Stimulation with an Artepillin C in Normoxia and Hypoxia Conditions

**Figure 2:**
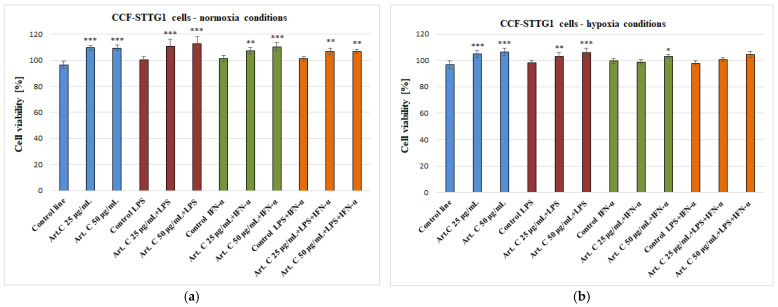
Cell viability [%] measured by MTT—CCF-STTG1 cells in combination with artepillin C (Art. C) with LPS, IFN-α, and LPS + IFN-α in normoxia (**a**) and hypoxia (**b**) conditions. The values represent the means ±SD of three independent assays: * *p* < 0.05, ** *p* < 0.01, and *** *p* < 0.001 compared to adequate control.

The CCF-STTG1 cells were incubated with artepillin C (25–50 μg/mL) for 24 h. Artepillin C, incubated under normoxia, increases the cell viability from 96.30 ± 2.71% to 109.54 ± 1.83% and 109.38 ± 2.16%, respectively (Figure 2a). Artepillin C, incubated under hypoxia, increases the cell viability from 96.73 ± 3.02% to 104.77 ± 2.71% and 106.47 ± 2.61%, respectively (Figure 2b). Artepillin C with LPS, IFN-α, or LPS + IFN-α has no effect on the decrease in viability of the cells tested in normoxia and hypoxia conditions (Figure 2a,b).

### 2.3. The Effect of Ethanolic Extract of Brazilian Green Propolis on Selected Pro-Inflammatory Cytokine Production by Astrocytes Cell Line CCF-STTG1 by LPS, IFN-α, and LPS + IFN-α in Normoxia and Hypoxia Conditions

**Figure 3:**
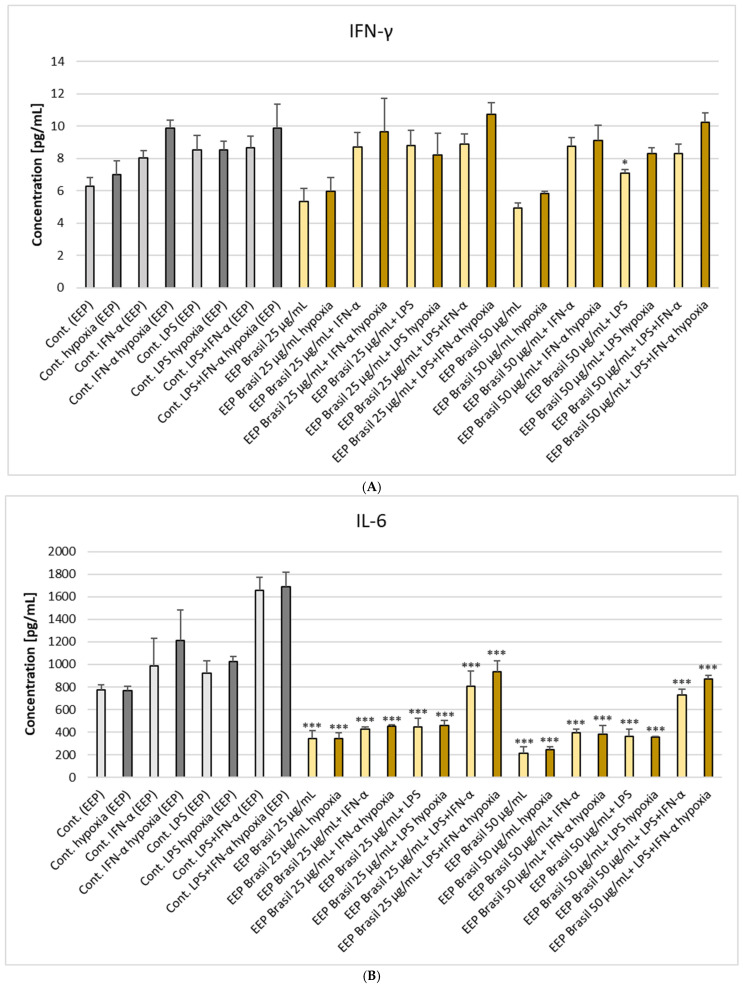

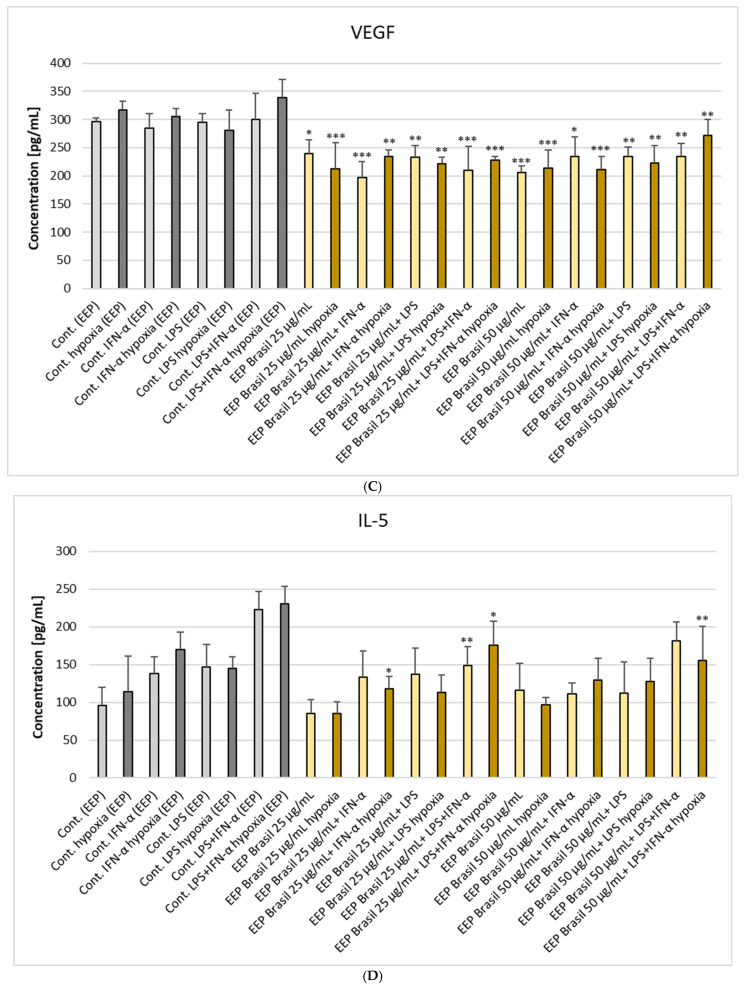

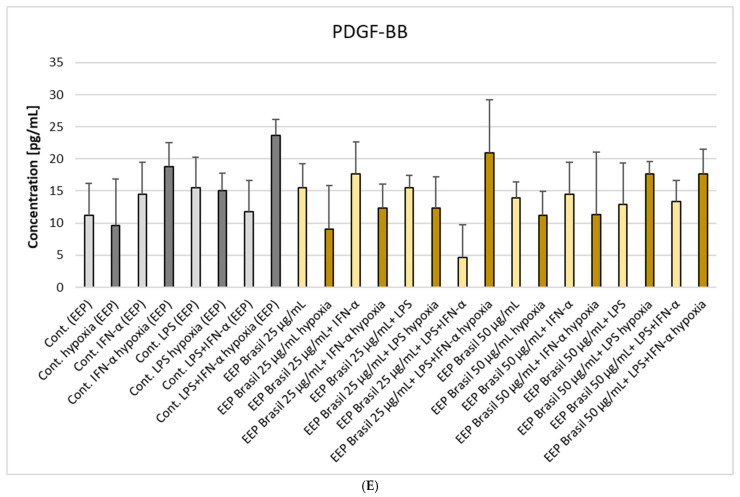
The effect of ethanolic extract of Brazilian green propolis (EEP-B) on selected cytokines: (**A**) IFN-γ, (**B**) IL-6, (**C**) VEGF, (**D**) IL-5, and (**E**) PDGF-BB production by native and stimulated astrocytes cell line CCF-STTG1 by LPS or/and IFN-α in normoxia and hypoxia conditions. Cells were incubated with EEP-B (25 and 50 μg/mL) for 24 h. Cytokine levels were measured using the Multiplex Bead-Based Cytokine Assay. Data represent the mean ± SD of three independent experiments: * *p* < 0.05, ** *p* < 0.01, and *** *p* < 0.001 (Fisher’s LSD test). Statistically significant reductions in IL-6 and VEGF suggest a potential anti-angiogenic effect of EEP-B. Additional data are available in the Supplementary Materials.

We examined the activity of ethanolic extract of Brazilian green propolis on the production of cytokines IL-6, IFN-γ, VEGF, and PDGF-BB by astrocytes present in the tumor CNS microenvironment. Additionally, we assessed the expression of IL-5, a cytokine produced by astrocytes but still poorly understood. The impact of EEP-B was investigated under experimental conditions involving LPS, IFN-α, and a combination of LPS with IFN-α. The results are presented in Figure 3 and the Supplementary Materials. Based on the existing literature and our previous study [23,24], it was predicted that LPS, IFN-α, and their combination would lead to increased secretion of the examined cytokines compared to the control cell line. EEP-B, at both tested concentrations and in all combinations, resulted in a decrease in IL-6 levels under both normoxic and hypoxic conditions (Figure 3). Specifically, for EEP-B alone, IL-6 levels decreased from 775.70 ± 42.78 pg/mL to 213.40 ± 57.98 pg/mL in normoxic conditions and from 767.58 ± 36.68 pg/mL to 248.25 ± 22.10 pg/mL in hypoxic conditions.

EEP-B (50 μg/mL) with LPS reduced the concentration of IFN-γ secretion by astrocytes from 8.55 ± 0.88 pg/mL to 7.10 ± 0.23 pg/mL only in normal conditions. EEP-B at 25 μg/mL with IFN-α and with LPS + IFN-α and also EEP-B at 50 μg/mL with LPS + IFN-α significantly reduced the concentration of IL-5 in hypoxia conditions to 118.41 ± 15.63 pg/mL, 175.41 ± 32.16 pg/mL, and 155.72 ± 45.03 pg/mL accordingly. In the case of VEGF, EEP-B at both concentrations used, alone and in combination with LPS and/or IFN-α, significantly reduced the concentration of this cytokine under both normoxia and hypoxia conditions (see Supplementary Materials). In the case of PDGF-BB, ethanolic extract of Brazilian green propolis did not statistically significantly alter the concentration of this labeled cytokine.

### 2.4. The Effect of Artepillin C on Selected Pro-Inflammatory Cytokine Production by Astrocyte Cell Line CCF-STTG1 by LPS, IFN-α, and LPS + IFN-α in Normoxia and Hypoxia Conditions

**Figure 4:**
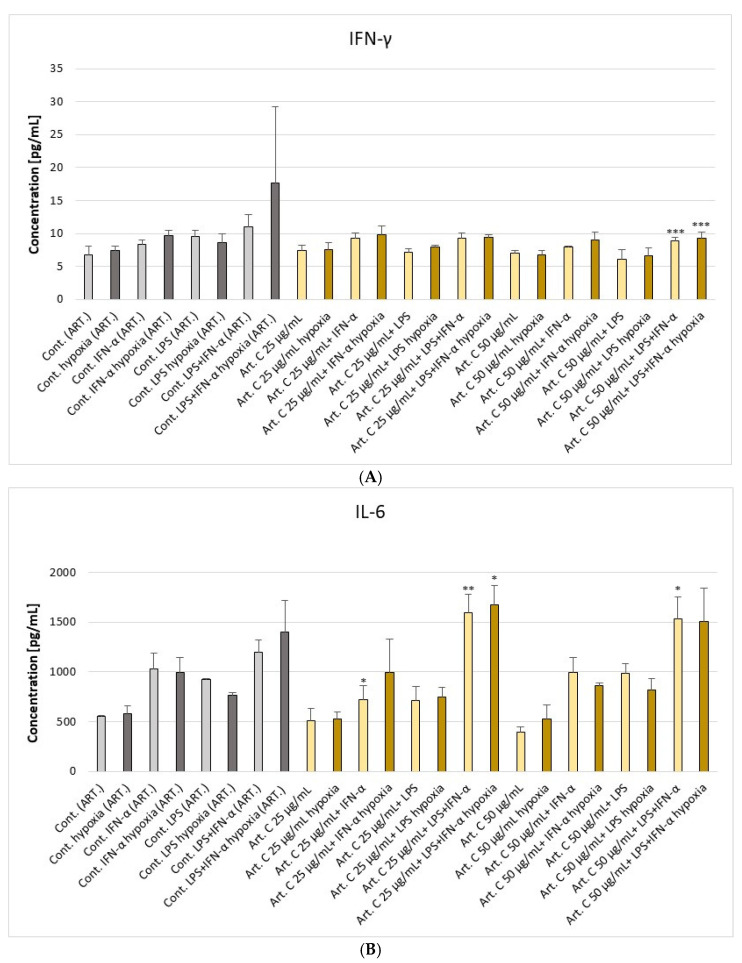

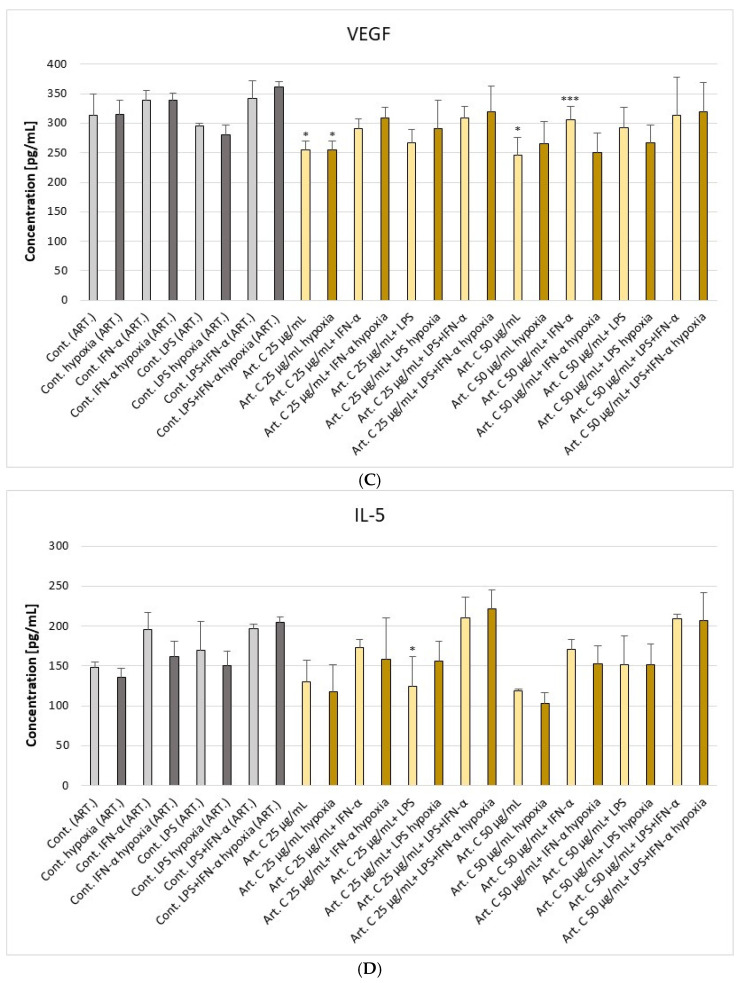

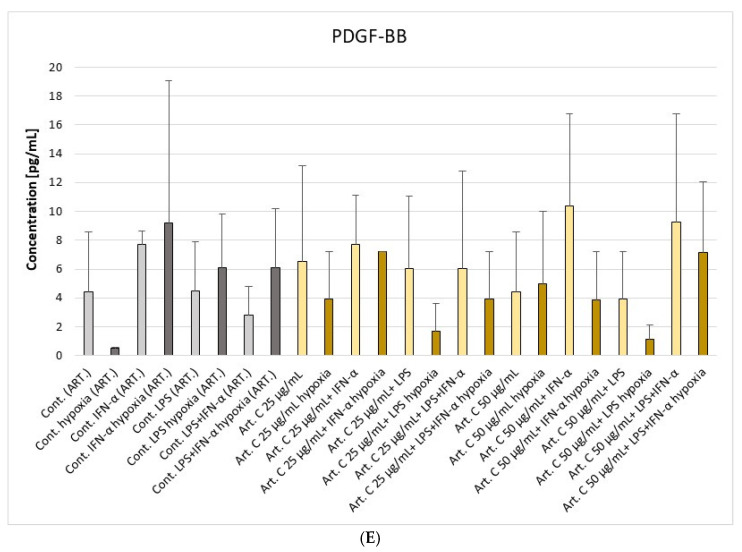
The effect of artepillin C (Art. C) on selected cytokines: (**A**) IFN-γ, (**B**) IL-6, (**C**) VEGF, (**D**) IL-5, and (**E**) PDGF-BB production by native and stimulated astrocyte cell line CCF-STTG1 by LPS or/and IFN-α in normoxia and hypoxia conditions. Cells were incubated with artepillin C (25 and 50 μg/mL) for 24 h. Cytokine levels were measured using the Multiplex Bead-Based Cytokine Assay. Data represent the mean ± SD of three independent experiments: * *p* < 0.05, ** *p* < 0.01, and *** *p* < 0.001 (Fisher’s LSD test). A significant reduction in IL-6 and VEGF levels was observed under certain conditions, suggesting a potential immunomodulatory effect of artepillin C. Additional data are available in Tables S11–S15.

Artepillin C at a concentration of 25 μg/mL, in combination with IFN-α, resulted in a statistically significant reduction in IL-6 concentrations, decreasing from 1029.94 pg/mL ± 159.68 pg/mL to 721.21 pg/mL ± 139.36 pg/mL in astrocyte secretions (Figure 4). However, at the same concentration, when combined additionally with LPS, artepillin C caused an increase in IL-6 levels to 1595.04 pg/mL ± 182.06 pg/mL. A further increase in IL-6 secretion was observed when astrocytes were stimulated with 25 μg/mL artepillin C in combination with LPS and/or IFN-α under hypoxic conditions, reaching 1675.91 ± 196.53 pg/mL. Artepillin C at a concentration of 50 μg/mL, in combination with LPS and IFN-α, caused a decrease in IFN-γ concentration under normoxic and hypoxic conditions to 8.88 pg/mL ± 0.53 pg/mL and 9.25 pg/mL ± 1.01 pg/mL, respectively. The concentration of IL-5, another cytokine tested, was reduced under the influence of artepillin C to 125.0 pg/mL ± 37.02 pg/mL. Decreased VEGF levels were observed after the incubation of astrocytes with 25 μg/mL artepillin C alone and in combination with IFN-γ (in normoxia). Artepillin C did not have a statistically significant effect on PDGF-BB release. 

### 2.5. Comparative Effect of EEP-B and Artepillin C on IL-6, IFN-γ, VEGF, PDGF-BB, and IL-5 Secretion in LPS and/or Induced by CCF-STTG1 Cell Lines

**Figure 5:**
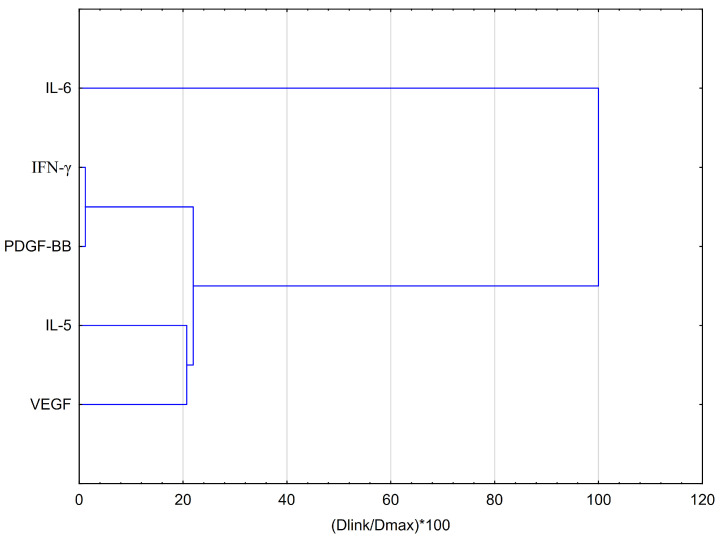
Dendrogram obtained via the HCA analysis of data regarding the behavior of IL-6, IFN-α, PDGF-BB, IL-5, and VEGF secretion by CCF-STTG1 astrocytes stimulated by LPS and/or IFN-α treatment with EEP-B. Dlink represents the distance between two clusters, also known as the linkage distance. Dmax refers to the greatest possible distance that can exist between clusters.

To evaluate the effects of EEP-B and its primary component, artepillin C, on the secretion of specific cytokines, hierarchical clustering analysis (HCA) and principal component analysis (PCA) were employed. HCA based on Euclidean distances (Figure 5) reveals that the data can be grouped into four distinct clusters. The second and fourth clusters show similar behavior between IFN-γ and PDGF-BB, as well as between IL-5 and VEGF. In contrast, IL-6 exhibits a different pattern, suggesting that EEP-B has a distinct impact on this cytokine. IL-6 is the most separated from the other clusters, indicating a unique effect or expression profile compared to the other cytokines analyzed. This suggests that IL-6 may serve as the dominant biomarker.

**Figure 6:**
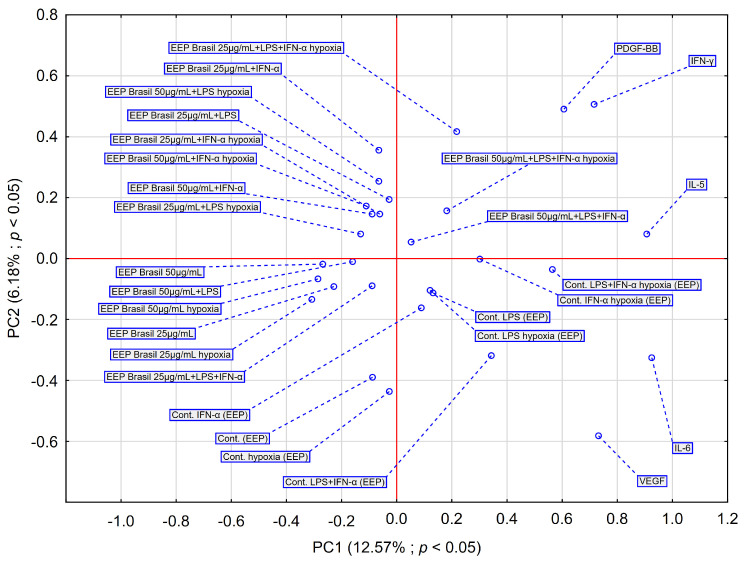
The PCA score plot depicts the data on the effect of EEP-B at various concentrations on the secretion of IL-6, IFN-α, PDGF-BB, IL-5, and VEGF by CCF-STTG1 astrocytes stimulated with LPS and/or IFN-α. Abbreviation: PC—principal component.

PCA score plots illustrating the effect of EEP-B on selected cytokines are presented in Figure 6. The insights from the PCA analysis of the EEP-B effect on selected markers are partially supported by the dendrogram generated through HCA analysis. PDGF-BB and IFN-α exhibit similar behaviors. PCA analysis showed that EEP-B at concentrations of 25 μg/mL and 50 μg/mL exhibited a strong similarity in behavior to EEP-B at the same concentrations under hypoxia. Similarly, EEP-B with LPS and/or IFN-α applied under normoxia behaved in a comparable manner under hypoxia. Under the influence of EEP-B, the examined cells release cytokines such as PDGF-BB and IFN-γ that exhibit similar behavior. IL-6 is crucial in PCA, and it is also the most relevant variable. This is confirmed by the result of the HCA analysis (Figure 5). Control cells under hypoxia showed increased levels of IL-6 and VEGF, suggesting cellular adaptation to hypoxia and a potential increase in angiogenic processes.

**Figure 7:**
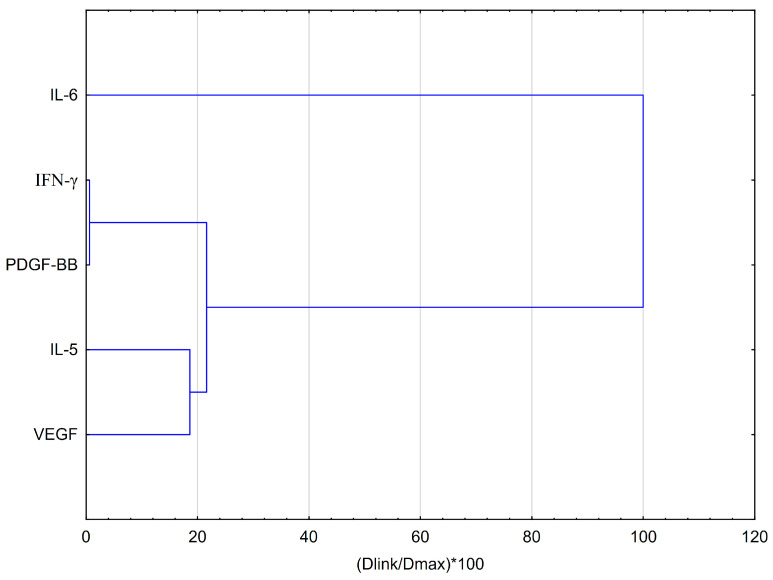
Dendrogram obtained via the HCA analysis of data regarding the behavior of IL-6, IFN-α, PDGF-BB, IL-5, and VEGF secretion by CCF-STTG1 astrocytes stimulated by LPS and/or IFN-α treatment with artepillin C. Dlink represents the distance between two clusters, also known as the linkage distance. Dmax refers to the greatest possible distance that can exist between clusters.

**Figure 8:**
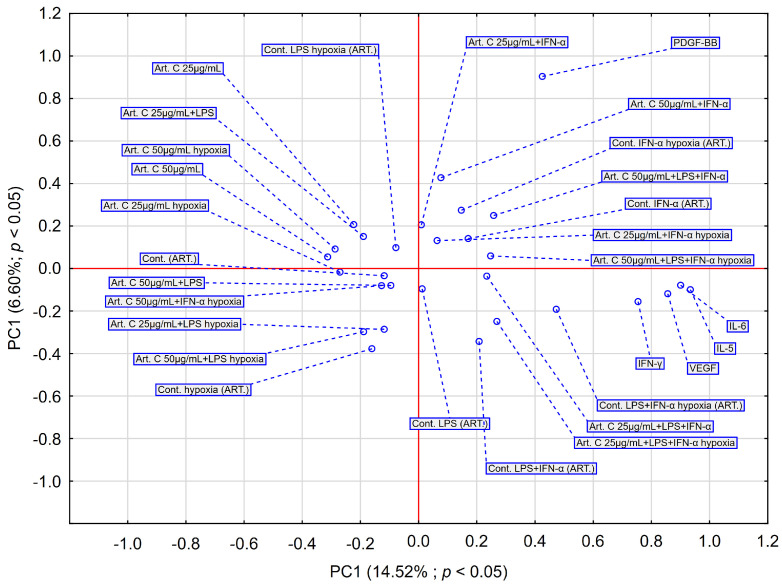
The PCA score plot depicts the data on the effect of artepillin C at various concentrations on the secretion of IL-6, IFN-α, PDGF-BB, IL-5, and VEGF by CCF-STTG1 astrocytes stimulated with LPS and/or IFN-α. Abbreviation: PC—principal component.

HCA analysis (Figure 7) of the effect of artepillin C on specific cytokines, based on Euclidean distances, reveals that the data can be grouped into four distinct clusters, similar to EEP-B. IL-6 remains the most distinct cytokine in both analyses. VEGF and IL-5 also group together, similar to their behavior with EEP-B, suggesting consistent regulation of these factors. The only difference with artepillin C is that the clusters are slightly farther apart, which may suggest greater variability in cytokine interactions compared to EEP-B. Cells under hypoxia conditions cluster in distinct regions of the graph, suggesting the effect of hypoxia on changes in relation to the cytokines being labeled. PDGF-BB is located in the upper right of the graph, indicating that its expression is specifically associated with higher concentrations of artepillin C (Figure 8). In contrast, IL-6, IL-5, and VEGF cluster in the lower-right part of the graph, suggesting their correlation. For artepillin C, PC1 explains a greater percentage of the variance (14.52%) than in the analysis for EEP-B (12.57%), suggesting that artepillin C has a stronger effect on sample separation.

## 3. Discussion

Historically, neurons were considered the most important components of the central nervous system. However, recent research has highlighted the critical role of non-neuronal cells in CNS function. These include macroglia—comprising lining cells, oligodendrocytes, and astrocytes—and microglia, which collectively support essential brain functions [25]. Astrocytes, the most abundant glial cells in the brain, play a crucial role in regulating the inflammatory response in the CNS. Under normal physiological conditions, IL-6 levels in the CNS are low. However, during brain injury, inflammation, or disease, IL-6 levels rise significantly [26]. High levels of hypoxia in tumor tissue play a significant role in the progression of malignant gliomas and other types of cancer while also increasing the resistance of tumor cells to radio- and chemotherapy. Propolis is a potentially effective anti-inflammatory agent capable of crossing the blood–brain barrier. In our study, we investigated the potential effects of Brazilian green propolis extract and artepillin C on astrocytes in grade IV malignancy.

In this study, the astrocyte cell line CCF-STTG1 produced IL-6 in response to LPS and/or IFN-α under both normal and hypoxia conditions. Ethanolic extract of Brazilian green propolis at both tested concentrations significantly reduced the concentration of this proinflammatory cytokine under both normoxic and hypoxic conditions. Interestingly, for artepillin C, no differences were observed in the decrease of IL-6 concentration, except for 25 μM artepillin C with IFN-α. Additionally, artepillin C combined with LPS and IFN-α significantly increased the concentration of IL-6. These observations suggest that the flavonoids in propolis may act synergistically compared to the single dominant compound. PCA analysis showed that the increase in EEP-B correlates with a marked decrease in IL-6 levels, particularly in the presence of LPS and IFN-α. A similar effect was observed for VEGF, though it is less pronounced. Wu Z. et al. demonstrated that Brazilian green propolis ethanol extract at 50 μg/mL significantly inhibited IL-1β, IL-6, and TNF-α in the microglial cell line MG6 under 1% O_2_ hypoxic exposure for 24 h [27]. Numerous studies have demonstrated a link between IL-6 expression, glioma progression, and patient survival outcomes. Higher levels of IL-6 mRNA expression were observed in glioma samples from patients with advanced histopathological stages compared to those with lower stages. Furthermore, IL-6 gene amplification was absent in low-grade and anaplastic tumors (0/17) but present in 15 out of 36 glioma specimens (42%). Notably, IL-6 gene amplification was associated with significantly shorter survival times compared to glioma patients without amplification. Immunohistochemical analysis also revealed IL-6 receptor expression in all glioblastoma multiforme samples (6/6, 100%), whereas it was absent in normal brain tissue (0/7, 0%) [28]. Many studies have shown that propolis inhibits and downregulates Myeloid differentiation primary response 88 (MyD88), interleukin-1 receptor-associated kinase 4 (IRAK4), and nuclear factor kappa-light-chain-enhancer of activated B cells (NF-κB), which is associated with the suppression of pro-inflammatory cytokine gene expression, including IL-6 and IFN-γ [29].

IFN-γ can facilitate tumor metastasis by inducing the expression of ICAM1 and CD13, promoting epithelial-mesenchymal transition (EMT), and stimulating the production of CXCR4 and MUC4. Additionally, IFN-γ enhances the synthesis of inhibitory molecules IDO (indoleamine-2,3-dioxygenase) and PD-L1 while inducing genome-wide immunoediting. High PD-L1 expression, stimulated by IFN-γ in lymphatic endothelial cells, prevents the migration of cytotoxic T lymphocytes to the tumor microenvironment (TME), thereby suppressing the immune response [30]. In our study, we demonstrated that EEP-B at 50 μg/mL with LPS can reduce the concentration of IFN-γ produced by astrocytes under normal conditions. However, artepillin C at 50 μg/mL with LPS and TNF-α reduced IFN-γ production in the examined cells under both conditions.

Vascular endothelial growth factor (VEGF) is a protein released in response to low oxygen levels that binds to receptor tyrosine kinases on endothelial cells, stimulating the formation of new blood vessels. VEGF levels are higher in glial tumors than in normal tissue [31,32]. In our study, we have shown that ethanolic extract of Brazilian green propolis (EEP-B) at 25 μg/mL and 50 μg/mL alone and with LPS and/or IFN-α significantly reduced the concentration of VEGF under normoxic and hypoxic conditions. Interestingly, artepillin C has shown a weaker effect compared to the reduction in the concentration of this cytokine. PDGF-BB is also a factor that plays an important role in the formation of blood vessels in the brain. Our study showed no statistically significant changes in PDGF-BB levels after stimulation of astrocytes with EEP-B and artepillin C, either alone or in combination with LPS/IFN-α.

Cytokine networks play a crucial role in the initiation and development of cancer. Therefore, in our study, we analyzed the cytokine profile released by astrocytes. There is no doubt that IL-5 is a cytokine involved in the pathogenesis of allergic inflammatory responses; however, its role in cancer processes remains poorly understood. In the central nervous system (CNS), IL-5 is produced by microglia and astrocytes. Liva et al. demonstrated that IL-5 induces microglial proliferation and affects nitric oxide metabolism and release. However, they did not detect the classical IL-5 receptor in microglia, suggesting that the effects of IL-5 on these cells are mediated by an unknown receptor [33]. In our study, we have shown that 25 μg/mL EEP-B in normoxia conditions and EEP-B at 25 μg/mL with IFN-α, IFN-α + LPS, and EEP-B at 50 μg/mL in combination with IFN-α + LPS can decrease the concentration of IL-5 secreted by astrocytes. Eiro N. et al. have shown that increased IL-5 expression in breast cancer is associated with higher rates of distant metastases and poor prognosis [34]. In turn, Lee E.J. et al. have demonstrated that IL-5 can directly promote the migration and invasion of bladder cancer cells through the activation of MAPK and Jak–Stat signaling pathways [35].

The altered expression of cytokines induces changes in the tumor microenvironment. These proteins act as intracellular mediators and contribute to host immunosuppression. Therefore, the impact of flavonoids and propolis, as a rich source of flavonoids, may be considered in cancer treatment. By influencing cytokines, these natural substances could complement standard cancer therapy, particularly immunotherapy [36]. In this study, the impact of propolis and artepillin C was investigated using two types of experimental models. Based on the literature data, hypoxia is a characteristic feature of solid tumors. Therefore, observations were conducted under both normoxic and hypoxic conditions. It is important to highlight that hypoxia is associated with the stimulation of angiogenesis, erythropoiesis, and alterations in cancer cell metabolism. Hypoxia promotes glycolysis in cancer cells while reducing mitochondrial respiration. Moreover, cancer cells exhibit increased de novo fatty acid synthesis. Given that hypoxia and the resulting metabolic changes in tumor cells are key therapeutic targets in oncology, studying the impact of immunomodulatory substances on the tumor microenvironment under hypoxic conditions may offer new strategies for immunotherapy. In particular, the effect of propolis and artepillin C on IL-6 levels under hypoxic conditions appears significant. Elevated IL-6 concentrations are linked to tumor growth stimulation. According to the cytokine field theory, the tumor microenvironment increases proinflammatory cytokine levels, such as IL-6, which may, in turn, induce nearby cells to produce additional cytokines [37]. As a result, excess cytokines are released into body fluids [38]. This elevated cytokine level in circulation also affects the immune and inflammatory responses associated with cancer.

Furthermore, hypoxia is closely correlated with angiogenesis. The proangiogenic cytokines identified in our study, including IL-6, IL-8, PDGF, and VEGF, play a key role in inducing angiogenesis and promoting tumor progression [39]. The highest concentrations of these cytokines, released by tumor cells, directly affect endothelial cells. Notably, the inhibitory effect of propolis on angiogenic cytokines appears to be significant. The most notable and statistically significant decrease in cytokine levels, especially IL-6 and VEGF, was observed after EEP-B treatment at both tested concentrations under both hypoxic and normoxic conditions. To conclude our study, the potential immunomodulatory effects of artepillin C and propolis on cytokine concentrations in the tumor microenvironment should be emphasized.

## 4. Materials and Methods

### 4.1. Preparation of EEP

Samples of Brazilian green propolis come from southeastern Brazil (the state of Minas Gerais). The raw Brazilian green propolis was collected by hand from beehives. The propolis sample was dried prior to processing. It was then extracted in 95% ethanol (*v*/*v*) for four days at 37 °C. Following extraction, the solution (EEP-B) was filtered using Whatman filter paper. We evaporated ethanol in a vacuum under reduced pressure at 60 °C. We obtained an ethanolic extract of Brazilian green propolis. EEP-B was subsequently dissolved in DMSO to achieve a final concentration of 50 mg/mL. In the medium, the final DMSO concentration was 0.1% (*v*/*v*) [40]. The identification and quantification of phenolic compounds in Brazilian green propolis extract were described previously using HPLC-DAD and UPLC-Q-TOF-MS methods [41].

### 4.2. CCF-STTG1 Cell Culture

Our experiments were conducted on an astrocyte cell line CCF-STTG1 provided by the American Type Culture Collection (ATCC, Manassas, VA, USA). These cells were isolated from the brain of a Caucasian 68-year-old female with grade IV astrocytoma. The CCF-STTG1 cells were grown at 37 °C in monolayer cultures in RPMI 1640, with 10% fetal bovine serum (FBS), supplemented with 100 U/mL penicillin and 10 μg/mL streptomycin solution. The cells were incubated in a humidified atmosphere at 37 °C with 5% CO_2_ and 95% air. The passages were conducted twice a week. The suspensions of 5 × 10^4^/mL cells were used for the experiments.

### 4.3. CCF-STTG1 Cell Stimulation with Brazilian Green Propolis or Artepillin C and/or LPS, IFN-α, and/or LPS + IFN-α

The next step of the study was to seed the cells in equivalent amounts of 200 µL per well into a 96-well plate for 48 h to obtain adhesion. After this time, the cells were treated with Brazilian green propolis at the final concentrations of 25 and 50 μg/mL and artepillin C at the final concentrations of 25 and 50 μg/mL, with or without LPS (200 ng/mL) and/or IFN-α (100 U). In parallel, after the addition of the compounds, the 96-well plate was placed in an incubator with 1% oxygen for 2 h to achieve a hypoxic condition. After this time, the plate was transferred to the standard incubator (5% CO_2_ and 95% air) for the remaining 22 h. All analyses were carried out in triplicate.

### 4.4. Cell Viability—MTT Cytotoxicity Assay

Cytotoxicity was measured using the 3-(4,5-dimethylthiazol-2-yl)-2,5-diphenyltetrazolium (MTT) assay as previously described [42]. EEP-B was added to 96-well plates at final concentrations of 25 and 50 μg/mL, with or without LPS, IFN-α, and LPS combined with IFN-α (each well contained 200 μL). After 24 h, the medium was removed and frozen at −80 °C for the next step of the experiment to determine cytokines. Then, 180 μL of medium and 20 μL of MTT solution (5 mg/mL in PBS) were added to each well and incubated for 4 h. The supernatants were then removed, and DMSO was added to each well to dissolve the formazan crystals. The absorbance was measured spectrophotometrically at a wavelength of 550 nm.

### 4.5. Multiplex Bead-Based Cytokine Assay

The Luminex Multiplex Immunoassay technique was used to determine the concentration of the cytokines studied. The technique uses color-coded (shades of red) sets of magnetic beads coated with antibodies specific for the cytokines to be determined. When added to supernatants and standards, the beads combine with specific analytes. The bead-linked cytokines are then detected by adding biotinylated antibodies, which react with a streptavidin-phycoerythrin conjugate. An ELx 50 magnetic washer (BioTek, USA) was used to wash the ferromagnetic beads after each incubation period. Quantitative determinations were performed using an xMAP Luminex Multiplex Immunoassay and the Multiplex Bead-Based Cytokine kit (Bio-Plex 3D Suspension Array System with Luminex xPonent Software, Version 4.3, Bio-Rad). This instrument operates on a flow cytometry-based technique utilizing two lasers: one to recognize the color of the beads (assigned to a specific analyte) and another to excite phycoerythrin to determine fluorescence, which is proportional to the amount of bound analyte. Concentrations of analytes were derived from curves constructed with the relevant standards.

### 4.6. Statistical Analysis

All analyses were conducted in triplicate, with results presented as averages. Statistical analyses were performed using STATISTICA 13.1 software (StatSoft Inc., Tulsa, OK, USA). The collected data were evaluated through hierarchical cluster analysis (full linkage with Euclidean distance) and principal component analysis (PCA). The PCA model was estimated using the NIPALS iterative algorithm, with a convergence threshold of 0.00001 and a maximum of 50 iterations. The number of components was determined by optimizing predictive performance through multiple cross-validations, with an upper limit set. The optimal PCA model was reduced to two components. The PCA analysis, illustrated in the PC1 vs. PC2 loadings chart, helped identify the variables with the greatest impact on the variability of the analyzed dataset and revealed the most significant correlations among them. These two classification methods, PCA and HCA, were applied to uncover natural groupings in the data and assess the differences in how EEP-B and artepillin C influence astrocytes. A one-way ANOVA was conducted to compare the effects of EEP-B and artepillin C at various concentrations on cytokine production stimulated by LPS, IFN-α, and their combination. Additionally, a *t*-test was used to analyze the MTT assay. A *p*-value of less than 0.05 was considered statistically significant.

## 5. Conclusions

Taking into consideration that elevated levels of IL-6 are associated with tumor growth, as well as elevated levels of VEGF being associated with angiogenesis stimulation, the observed impact of propolis on these cytokines demonstrates that propolis may potentially inhibit tumor progression. However, neither EEP-B nor artepillin C influenced PDGF-BB levels. These results indicate that Brazilian green propolis could act as a potential immunomodulator in combination therapies for gliomas of different malignancy grades. We need further studies, but it is evident that propolis, as an agent that modifies the immune response, may be a potential alternative to support classical therapies for cancer.

## Data Availability

Data are contained within the article.

## Supplementary Materials

The following supporting information can be downloaded at https://www.mdpi.com/article/10.3390/ph18030389/s1. Table S1. The effect of ethanolic extract of Brazilian green propolis with LPS and/or IFN-α on the production of IL-6 compared to control in CCF-STTG1 cells (*n* = 3) in normoxia. Fisher’s LSD test was used to evaluate statistical significance. Results marked in red are statistically significant in Fisher’s LSD test. Multivariate Tests of Significance (F = 6.009, *p* < 0.05). Table S2. The effect of ethanolic extract of Brazilian green propolis with LPS and/or IFN-α on the production of IFN-γ compared to control in CCF-STTG1 cells (*n* = 3) in normoxia. Fisher’s LSD test was used to evaluate statistical significance. Results marked in red are statistically significant in Fisher’s LSD test. Multivariate Tests of Significance (F = 6.009, *p* < 0.05). Table S3. The effect of ethanolic extract of Brazilian green propolis with LPS and/or IFN-α on the production of IL-5 compared to control in CCF-STTG1 cells (*n* = 3) in normoxia. Fisher’s LSD test was used to evaluate statistical significance. Results marked in red are statistically significant in Fisher’s LSD test. Multivariate Tests of Significance (F = 6.009, *p* < 0.05). Table S4. The effect of ethanolic extract of Brazilian green propolis with LPS and/or IFN-α on the production of VEGF compared to control in CCF-STTG1 cells (*n* = 3) in normoxia. Fisher’s LSD test was used to evaluate statistical significance. Results marked in red are statistically significant in Fisher’s LSD test. Multivariate Tests of Significance (F = 6.009, *p* < 0.05). Table S5. The effect of ethanolic extract of Brazilian green propolis with LPS and/or IFN-α on the production of PDGF-BB in compared to control in CCF-STTG1 cells (*n* = 3) in normoxia. Fisher’s LSD test was used to evaluate statistical significance. Results marked in red are statistically significant in Fisher’s LSD test. Multivariate Tests of Significance (F = 6.009, *p* < 0.05). Table S6. The effect of ethanolic extract of Brazilian green propolis with LPS and/or IFN-α on the production of IL-6 compared to control in CCF-STTG1 cells (*n* = 3) in hypoxia. Fisher’s LSD test was used to evaluate statistical significance. Results marked in red are statistically significant in Fisher’s LSD test. Multivariate Tests of Significance (F = 4.363, *p* < 0.05). Table S7. The effect of ethanolic extract of Brazilian green propolis with LPS and/or IFN-α on the production of IFN-γ compared to control in CCF-STTG1 cells (*n* = 3) in hypoxia. Fisher’s LSD test was used to evaluate statistical significance. Results marked in red are statistically significant in Fisher’s LSD test. Multivariate Tests of Significance (F = 4.363, *p* < 0.05). Table S8. The effect of ethanolic extract of Brazilian green propolis with LPS and/or IFN-α on the production of IL-5 compared to control in CCF-STTG1 cells (*n* = 3) in hypoxia. Fisher’s LSD test was used to evaluate statistical significance. Results marked in red are statistically significant in Fisher’s LSD test. Multivariate Tests of Significance (F = 4.363, *p* < 0.05). Table S9. The effect of ethanolic extract of Brazilian green propolis with LPS and/or IFN-α on the production of VEGF compared to control in CCF-STTG1 cells (*n* = 3) in hypoxia. Fisher’s LSD test was used to evaluate statistical significance. Results marked in red are statistically significant in Fisher’s LSD test. Multivariate Tests of Significance (F = 4.363, *p* < 0.05). Table S10. The effect of ethanolic extract of Brazilian green propolis with LPS and/or IFN-α on the production of PDGF-BB compared to control in CCF-STTG1 cells (*n* = 3) in hypoxia. Fisher’s LSD test was used to evaluate statistical significance. Results marked in red are statistically significant in Fisher’s LSD test. Multivariate Tests of Significance (F = 4.363, *p* < 0.05). Table S11. The effect of artepillin C with LPS and/or IFN-α on the production of IL-6 compared to control in CCF-STTG1 cells (*n* = 3) in normoxia. Fisher’s LSD test was used to evaluate statistical significance. Results marked in red are statistically significant in Fisher’s LSD test. Multivariate Tests of Significance (F = 4.495, *p* < 0.05). Table S12. The effect of artepillin C with LPS and/or IFN-α on the production of IFN-γ compared to control in CCF-STTG1 cells (*n* = 3) in normoxia. Fisher’s LSD test was used to evaluate statistical significance. Results marked in red are statistically significant in Fisher’s LSD test. Multivariate Tests of Significance (F = 4.495, *p* < 0.05). Table S13. The effect of artepillin C with LPS and/or IFN-α on the production of IL-5 compared to control in CCF-STTG1 cells (*n* = 3) in normoxia. Fisher’s LSD test was used to evaluate statistical significance. Results marked in red are statistically significant in Fisher’s LSD test. Multivariate Tests of Significance (F = 4.495, *p* < 0.05). Table S14. The effect of artepillin C with LPS and/or IFN-α on the production of VEGF compared to control in CCF-STTG1 cells (*n* = 3) in normoxia. Fisher’s LSD test was used to evaluate statistical significance. Results marked in red are statistically significant in Fisher’s LSD test. Multivariate Tests of Significance (F = 4.495, *p* < 0.05). Table S15. The effect of artepillin C with LPS and/or IFN-α on the production of PDGF-BB compared to control in CCF-STTG1 cells (*n* = 3) in normoxia. Fisher’s LSD test was used to evaluate statistical significance. Results marked in red are statistically significant in Fisher’s LSD test. Multivariate Tests of Significance (F = 4.495, *p* < 0.05). Table S16. The effect of artepillin C with LPS and/or IFN-α on the production of IL-6 compared to control in CCF-STTG1 cells (*n* = 3) in hypoxia. Fisher’s LSD test was used to evaluate statistical significance. Results marked in red are statistically significant in Fisher’s LSD test. Multivariate Tests of Significance (F = 2.5011, *p* < 0.05). Table S17. The effect of artepillin C with LPS and/or IFN-α on the production of IL-6 compared to control in CCF-STTG1 cells (*n* = 3) in hypoxia. Fisher’s LSD test was used to evaluate statistical significance. Results marked in red are statistically significant in Fisher’s LSD test. Multivariate Tests of Significance (F = 2.5011, *p* < 0.05). Table S18. The effect of artepillin C with LPS and/or IFN-α on the production of IL-5 compared to control in CCF-STTG1 cells (*n* = 3) in hypoxia. Fisher’s LSD test was used to evaluate statistical significance. Results marked in red are statistically significant in Fisher’s LSD test. Multivariate Tests of Significance (F = 2.5011, *p* < 0.05). Table S19. The effect of artepillin C with LPS and/or IFN-α on the production of VEGF compared to control in CCF-STTG1 cells (*n* = 3) in hypoxia. Fisher’s LSD test was used to evaluate statistical significance. Results marked in red are statistically significant in Fisher’s LSD test. Multivariate Tests of Significance (F = 2.5011, *p* < 0.05). Table S20. The effect of artepillin C with LPS and/or IFN-α on the production of PDGF-BB compared to control in CCF-STTG1 cells (*n* = 3) in hypoxia. Fisher’s LSD test was used to evaluate statistical significance. Results marked in red are statistically significant in Fisher’s LSD test. Multivariate Tests of Significance (F = 2.5011, *p* < 0.05).

## Abbreviations

The following abbreviations are used in this manuscript:

CNS	Central Nervous System
LPS	lipopolysaccharide
IL-6	interleukin 6
IFN-γ	interferon gamma
VEGF	vascular endothelial growth factor
PDGF-BB	platelet-derived growth factor
