# Supplementary material for: The Effect of Ethanolic Extract of Brazilian Green Propolis and Artepillin C on Cytokine Secretion by Stage IV Glioma Cells Under Hypoxic and Normoxic Conditions
Source: Pharmaceuticals (Basel). 2025 Mar 9;18(3):389. doi: 10.3390/ph18030389 (PMC11944379; doi:10.3390/ph18030389)
Supplement: Supplementary file 1 [file pharmaceuticals-18-00389-s001.zip › pharmaceuticals-3504934-supplementary.pdf]

Supplementary Materials

The Effect of Ethanolic Extract of Brazilian Green Propolis and Artepillin C on Cytokine Secretion by Stage IV Glioma Cells Under Hypoxic and Normoxic Conditions

Małgorzata Kłósek [1,*], Anna Kurek-Górecka [1], Radosław Balwierz [2], Grażyna Pietsz [1] and Zenon P. Czuba [1]

[1] Department of Microbiology and Immunology, Faculty of Medical Sciences, Medical University of Silesia in Katowice, Jordana 19, 41-808 Zabrze, Poland; akurekgorecka@sum.edu.pl (A.K.-G.); gpietsz@sum.edu.pl (G.P.); zczuba@sum.edu.pl (Z.P.C.)
[2] Institute of Chemistry, University of Opole, Oleska 48, 45-052 Opole, Poland; radoslaw.balwierz@uni.opole.pl
* Correspondence: mklosek@sum.edu.pl; Tel./Fax: +48-322-722-554

Academic Editor: Luis Apaza Ticona

Received: 17 February 2025
Revised: 5 March 2025
Accepted: 7 March 2025
Published: 9 March 2025

Citation: Kłósek, M.; Kurek-Górecka, A.; Balwierz, R.; Pietsz, G.; Czuba, Z.P. The Effect of Ethanolic Extract of Brazilian Green Propolis and Artepillin C on Cytokine Secretion by Stage Iv Glioma Cells Under Hypoxic and Normoxic Conditions. *Pharmaceuticals* **2025**, *18*, 389. https://doi.org/10.3390/ph18030389

Copyright: © 2025 by the author. Licensee MDPI, Basel, Switzerland. This article is an open access article distributed under the terms and conditions of the Creative Commons Attribution (CC BY) license (https://creativecommons.org/licenses/by/4.0/).

Content

Table S1. The effect of etanolic extract of Brazilian green propolis with LPS and/or IFN-α on the production of IL-6 in compared to control in CCF-STTG1 cells (n=3) in normoxia. Fisher's LSD test was used to evaluate statistical significance. Results marked in red are statistically significant in Fisher's LSD test. Multivariate Tests of Significance ($F = 6.009$, $p < 0.05$).

Table S2. The effect of etanolic extract of Brazilian green propolis with LPS and/or IFN-α on the production of IFN-γ in compared to control in CCF-STTG1 cells (n=3) in normoxia. Fisher's LSD test was used to evaluate statistical significance. Results marked in red are statistically significant in Fisher's LSD test. Multivariate Tests of Significance ($F = 6.009$, $p < 0.05$).

Table S3. The effect of etanolic extract of Brazilian green propolis with LPS and/or IFN-α on the production of IL-5 in compared to control in CCF-STTG1 cells (n=3) in normoxia. Fisher's LSD test was used to evaluate statistical significance. Results marked in red are statistically significant in Fisher's LSD test. Multivariate Tests of Significance ($F = 6.009$, $p < 0.05$).

Table S4. The effect of etanolic extract of Brazilian green propolis with LPS and/or IFN-α on the production of VEGF in compared to control in CCF-STTG1 cells (n=3) in normoxia. Fisher's LSD test was used to evaluate statistical significance. Results marked in red are statistically significant in Fisher's LSD test. Multivariate Tests of Significance ($F = 6.009$, $p < 0.05$).

Table S5. The effect of etanolic extract of Brazilian green propolis with LPS and/or IFN-α on the production of PDGF-BB in compared to control in CCF-STTG1 cells (n=3) in normoxia. Fisher's LSD test was used to evaluate statistical significance. Results marked in red are statistically significant in Fisher's LSD test. Multivariate Tests of Significance ($F = 6.009$, $p < 0.05$).

Table S6. The effect of etanolic extract of Brazilian green propolis with LPS and/or IFN-α on the production of IL-6 in compared to control in CCF-STTG1 cells (n=3) in hypoxia. Fisher's LSD test was used to evaluate statistical significance. Results marked in red are statistically significant in Fisher's LSD test. Multivariate Tests of Significance ($F = 4.363$, $p < 0.05$).

Table S7. The effect of etanolic extract of Brazilian green propolis with LPS and/or IFN-α on the production of IFN-γ in compared to control in CCF-STTG1 cells (n=3) in hypoxia. Fisher's LSD test was used to evaluate statistical significance. Results marked in red are statistically significant in Fisher's LSD test. Multivariate Tests of Significance ($F = 4.363$, $p < 0.05$).

Table S8. The effect of etanolic extract of Brazilian green propolis with LPS and/or IFN-α on the production of IL-5 in compared to control in CCF-STTG1 cells (n=3) in hypoxia. Fisher's LSD test was used to evaluate statistical significance. Results marked in red are statistically significant in Fisher's LSD test. Multivariate Tests of Significance ($F = 4.363$, $p < 0.05$).

Table S9. The effect of etanolic extract of Brazilian green propolis with LPS and/or IFN-α on the production of VEGF in compared to control in CCF-STTG1 cells (n=3) in hypoxia. Fisher's LSD test was used to evaluate statistical significance. Results marked in red are statistically significant in Fisher's LSD test. Multivariate Tests of Significance ($F = 4.363$, $p < 0.05$).

Table S10. The effect of etanolic extract of Brazilian green propolis with LPS and/or IFN-α on the production of PDGF-BB in compared to control in CCF-STTG1 cells (n=3) in hypoxia. Fisher's LSD test was used to evaluate statistical significance. Results marked in red are statistically significant in Fisher's LSD test. Multivariate Tests of Significance ($F = 4.363$, $p < 0.05$).

Table S11. The effect of artepilin C with LPS and/or IFN-α on the production of IL-6 in compared to control in CCF-STTG1 cells (n=3) in normoxia. Fisher's LSD test was used to evaluate statistical significance. Results marked in red are statistically significant in Fisher's LSD test. Multivariate Tests of Significance ($F = 4.495$, $p < 0.05$).

Table S12. The effect of artepilin C with LPS and/or IFN-α on the production of IFN-γ in compared to control in CCF-STTG1 cells (n=3) in normoxia. Fisher's LSD test was used to evaluate statistical significance. Results marked in red are statistically significant in Fisher's LSD test. Multivariate Tests of Significance ($F = 4.495$, $p < 0.05$).

Table S13. The effect of artepilin C with LPS and/or IFN-α on the production of IL-5 in compared to control in CCF-STTG1 cells (n=3) in normoxia. Fisher's LSD test was used to evaluate statistical significance. Results marked in red are statistically significant in Fisher's LSD test. Multivariate Tests of Significance ($F = 4.495$, $p < 0.05$).

Table S14. The effect of artepilin C with LPS and/or IFN-α on the production of VEGF in compared to control in CCF-STTG1 cells (n=3) in normoxia. Fisher's LSD test was used to evaluate statistical significance. Results marked in red are statistically significant in Fisher's LSD test. Multivariate Tests of Significance ($F = 4.495$, $p < 0.05$).

Table S15. The effect of artepilin C with LPS and/or IFN-α on the production of PDGF-BB in compared to control in CCF-STTG1 cells (n=3) in normoxia. Fisher's LSD test was used to evaluate statistical significance. Results marked in red are statistically significant in Fisher's LSD test. Multivariate Tests of Significance ($F = 4.495$, $p < 0.05$).

Table S16. The effect of artepilin C with LPS and/or IFN-α on the production of IL-6 in compared to control in CCF-STTG1 cells (n=3) in hypoxia. Fisher's LSD test was used to evaluate statistical significance. Results marked in red are statistically significant in Fisher's LSD test. Multivariate Tests of Significance ($F = 2.5011$, $p < 0.05$).

Table S17. The effect of artepilin C with LPS and/or IFN-α on the production of IL-6 in compared to control in CCF-STTG1 cells (n=3) in hypoxia. Fisher's LSD test was used to evaluate statistical significance. Results marked in red are statistically significant in Fisher's LSD test. Multivariate Tests of Significance ($F = 2.5011$, $p < 0.05$).

Table S18. The effect of artepilin C with LPS and/or IFN-α on the production of IL-5 in compared to control in CCF-STTG1 cells (n=3) in hypoxia. Fisher's LSD test was used to evaluate statistical significance. Results marked in red are statistically significant in Fisher's LSD test. Multivariate Tests of Significance ($F = 2.5011$, $p < 0.05$).

Table S19. The effect of artepilin C with LPS and/or IFN-α on the production of VEGF in compared to control in CCF-STTG1 cells (n=3) in hypoxia. Fisher's LSD test was used to evaluate statistical significance. Results marked in red are statistically significant in Fisher's LSD test. Multivariate Tests of Significance ($F = 2.5011$, $p < 0.05$).

Table S20. The effect of artepilin C with LPS and/or IFN-α on the production of PDGF-BB in compared to control in CCF-STTG1 cells (n=3) in hypoxia. Fisher's LSD test was used to evaluate statistical significance. Results marked in red are statistically significant in Fisher's LSD test. Multivariate Tests of Significance ($F = 2.5011$, $p < 0.05$).

Table S1. The effect of etanolic extract of Brazilian green propolis with LPS and/or IFN-α on the production of IL-6 in compared to control in CCF-STTG1 cells (n=3) in normoxia. Fisher's LSD test was used to evaluate statistical significance. Results marked in red are statistically significant in Fisher's LSD test. Multivariate Tests of Significance ($F = 6.009$, $p < 0.05$).

	LSD test; variable IL-6<br>Probabilities for Post Hoc Tests<br>Error: Between MS = 10590,, df = 24,000												
Cell No.	Sample	{1}	{2}	{3}	{4}	{5}	{6}	{7}	{8}	{9}	{10}	{11}	{12}
1	EEP Brasil 25μg/ml		0,344263	0,245329	0,000012	0,132616	0,564840	0,855444	0,000119	0,000030	0,000000	0,000000	0,000000
2	EEP Brasil 25μg/ml+ IFN-γ	0,344263		0,823026	0,000138	0,018731	0,706508	0,442635	0,001366	0,000342	0,000001	0,000004	0,000000
3	EEP Brasil 25μg/ml+ LPS	0,245329	0,823026		0,000245	0,011207	0,549433	0,324086	0,002390	0,000606	0,000001	0,000007	0,000000
4	EEP Brasil 25μg/ml+ LPS+IFN-γ	0,000012	0,000138	0,000245		0,000000	0,000052	0,000019	0,372993	0,723681	0,040695	0,173838	0,000000
5	EEP Brasil 50μg/ml	0,132616	0,018731	0,011207	0,000000		0,042680	0,094506	0,000002	0,000001	0,000000	0,000000	0,000000
6	EEP Brasil 50μg/ml+ IFN-γ	0,564840	0,706508	0,549433	0,000052	0,042680		0,692989	0,000525	0,000130	0,000000	0,000002	0,000000
7	EEP Brasil 50μg/ml+ LPS	0,855444	0,442635	0,324086	0,000019	0,094506	0,692989		0,000190	0,000047	0,000000	0,000001	0,000000
8	EEP Brasil 50μg/ml+ LPS+IFN-γ	0,000119	0,001366	0,002390	0,372993	0,000002	0,000525	0,000190		0,587325	0,005237	0,029838	0,000000
9	Cont. (EEP)	0,000030	0,000342	0,000606	0,723681	0,000001	0,000130	0,000047	0,587325		0,018753	0,091269	0,000000
10	Cont. IFN-γ (EEP)	0,000000	0,000001	0,000001	0,040695	0,000000	0,000000	0,000000	0,005237	0,018753		0,453638	0,000000
11	Cont. LPS (EEP)	0,000000	0,000004	0,000007	0,173838	0,000000	0,000002	0,000001	0,029838	0,091269	0,453638		0,000000
12	Cont. LPS+IFN-γ (EEP)	0,000000	0,000000	0,000000	0,000000	0,000000	0,000000	0,000000	0,000000	0,000000	0,000000	0,000000	

 https://doi.org/10.3390/ph18030389

Table S2. The effect of etanolic extract of Brazilian green propolis with LPS and/or IFN-α on the production of IFN-γ in compared to control in CCF-STTG1 cells (n=3) in normoxia. Fisher's LSD test was used to evaluate statistical significance. Results marked in red are statistically significant in Fisher's LSD test. Multivariate Tests of Significance ($F = 6.009$, $p < 0.05$).

	LSD test; variable IFN-γ<br>Probabilities for Post Hoc Tests<br>Error: Between MS = ,45110, df = 24,000												
Cell No.	Sample	{1}	{2}	{3}	{4}	{5}	{6}	{7}	{8}	{9}	{10}	{11}	{12}
1	EEP Brasil 25μg/ml		0,000002	0,000001	0,000001	0,507416	0,000002	0,003445	0,000014	0,094290	0,000048	0,000004	0,000002
2	EEP Brasil 25μg/ml+ IFN-γ	0,000002		0,845162	0,725347	0,000000	0,906565	0,007449	0,460488	0,000179	0,232845	0,784618	0,968921
3	EEP Brasil 25μg/ml+ LPS	0,000001	0,845162		0,875743	0,000000	0,937845	0,004655	0,352820	0,000108	0,168069	0,639921	0,814829
4	EEP Brasil 25μg/ml+ LPS+IFN-γ	0,000001	0,725347	0,875743		0,000000	0,814786	0,003176	0,279877	0,000072	0,127328	0,533457	0,696452
5	EEP Brasil 50μg/ml	0,507416	0,000000	0,000000	0,000000		0,000000	0,000648	0,000003	0,023714	0,000009	0,000001	0,000000
6	EEP Brasil 50μg/ml+ IFN-γ	0,000002	0,906565	0,937845	0,814786	0,000000		0,005623	0,393608	0,000132	0,191968	0,696328	0,875787
7	EEP Brasil 50μg/ml+ LPS	0,003445	0,007449	0,004655	0,003176	0,000648	0,005623		0,039900	0,145958	0,102273	0,014134	0,008171
8	EEP Brasil 50μg/ml+ LPS+IFN-γ	0,000014	0,460488	0,352820	0,279877	0,000003	0,393608	0,039900		0,001191	0,639874	0,639988	0,484105
9	Cont. (EEP)	0,094290	0,000179	0,000108	0,000072	0,023714	0,000132	0,145958	0,001191		0,003826	0,000361	0,000198
10	Cont. IFN-γ (EEP)	0,000048	0,232845	0,168069	0,127328	0,000009	0,191968	0,102273	0,639874	0,003826		0,352788	0,247776
11	Cont. LPS (EEP)	0,000004	0,784618	0,639921	0,533457	0,000001	0,696328	0,014134	0,639988	0,000361	0,352788		0,814656
12	Cont. LPS+IFN-γ (EEP)	0,000002	0,968921	0,814829	0,696452	0,000000	0,875787	0,008171	0,484105	0,000198	0,247776	0,814656	

Table S3. The effect of etanolic extract of Brazilian green propolis with LPS and/or IFN-α on the production of IL-5 in compared to control in CCF-STTG1 cells (n=3) in normoxia. Fisher's LSD test was used to evaluate statistical significance. Results marked in red are statistically significant in Fisher's LSD test. Multivariate Tests of Significance ($F = 6.009$, $p < 0.05$).

	LSD test; variable IL-5<br>Probabilities for Post Hoc Tests<br>Error: Between MS = 814,82, df = 24,000												
Cell No.	Sample	{1}	{2}	{3}	{4}	{5}	{6}	{7}	{8}	{9}	{10}	{11}	{12}
1	EEP Brasil 25μg/ml		0,050896	0,035364	0,012199	0,203066	0,277630	0,256608	0,000390	0,648621	0,031191	0,014983	0,000004
2	EEP Brasil 25μg/ml+ IFN-γ	0,050896		0,862756	0,518300	0,462538	0,354400	0,380677	0,050028	0,124076	0,817295	0,576959	0,000766
3	EEP Brasil 25μg/ml+ LPS	0,035364	0,862756		0,634945	0,366030	0,274180	0,296225	0,071049	0,089689	0,953563	0,699389	0,001187
4	EEP Brasil 25μg/ml+ LPS+IFN-γ	0,012199	0,518300	0,634945		0,173640	0,122686	0,134536	0,171954	0,033933	0,676744	0,928962	0,003879
5	EEP Brasil 50μg/ml	0,203066	0,462538	0,366030	0,173640		0,844928	0,884783	0,009695	0,405293	0,336762	0,201876	0,000115
6	EEP Brasil 50μg/ml+ IFN-γ	0,277630	0,354400	0,274180	0,122686	0,844928		0,959540	0,006089	0,522243	0,250373	0,144126	0,000070
7	EEP Brasil 50μg/ml+ LPS	0,256608	0,380677	0,296225	0,134536	0,884783	0,959540		0,006876	0,490247	0,271023	0,157632	0,000079
8	EEP Brasil 50μg/ml+ LPS+IFN-γ	0,000390	0,050028	0,071049	0,171954	0,009695	0,006089	0,006876		0,001246	0,079691	0,147149	0,086424
9	Cont. (EEP)	0,648621	0,124076	0,089689	0,033933	0,405293	0,522243	0,490247	0,001246		0,080111	0,041039	0,000014
10	Cont. IFN-γ (EEP)	0,031191	0,817295	0,953563	0,676744	0,336762	0,250373	0,271023	0,079691	0,080111		0,742802	0,001375
11	Cont. LPS (EEP)	0,014983	0,576959	0,699389	0,928962	0,201876	0,144126	0,157632	0,147149	0,041039	0,742802		0,003116
12	Cont. LPS+IFN-γ (EEP)	0,000004	0,000766	0,001187	0,003879	0,000115	0,000070	0,000079	0,086424	0,000014	0,001375	0,003116	

Table S4. The effect of etanolic extract of Brazilian green propolis with LPS and/or IFN-α on the production of VEGF in compared to control in CCF-STTG1 cells (n=3) in normoxia. Fisher's LSD test was used to evaluate statistical significance. Results marked in red are statistically significant in Fisher's LSD test. Multivariate Tests of Significance ($F = 6.009$, $p < 0.05$).

	LSD test; variable VEGF<br>Probabilities for Post Hoc Tests<br>Error: Between MS = 736,50, df = 24,000												
Cell No.	Sample	{1}	{2}	{3}	{4}	{5}	{6}	{7}	{8}	{9}	{10}	{11}	{12}
1	EEP Brasil 25µg/ml		0,059632	0,773097	0,184326	0,140883	0,817679	0,809280	0,802663	0,019321	0,052767	0,019566	0,012431
2	EEP Brasil 25µg/ml+ IFN-γ	0,059632		0,104860	0,547487	0,653707	0,093959	0,095923	0,097499	0,000154	0,000508	0,000156	0,000094
3	EEP Brasil 25µg/ml+ LPS	0,773097	0,104860		0,292934	0,230186	0,953815	0,962454	0,969280	0,009946	0,028596	0,010077	0,006291
4	EEP Brasil 25µg/ml+ LPS+IFN-γ	0,184326	0,547487	0,292934		0,877431	0,268069	0,272603	0,276223	0,000723	0,002332	0,000733	0,000442
5	EEP Brasil 50µg/ml	0,140883	0,653707	0,230186	0,877431		0,209445	0,213214	0,216228	0,000488	0,001587	0,000495	0,000298
6	EEP Brasil 50µg/ml+ IFN-γ	0,817679	0,093959	0,953815	0,268069	0,209445		0,991348	0,984517	0,011388	0,032431	0,011538	0,007226
7	EEP Brasil 50µg/ml+ LPS	0,809280	0,095923	0,962454	0,272603	0,213214	0,991348		0,993167	0,011104	0,031679	0,011250	0,007041
8	EEP Brasil 50µg/ml+ LPS+IFN-γ	0,802663	0,097499	0,969280	0,276223	0,216228	0,984517	0,993167		0,010884	0,031096	0,011027	0,006899
9	Cont. (EEP)	0,019321	0,000154	0,009946	0,000723	0,000488	0,011388	0,011104	0,010884		0,642496	0,995536	0,847045
10	Cont. IFN-γ (EEP)	0,052767	0,000508	0,028596	0,002332	0,001587	0,032431	0,031679	0,031096	0,642496		0,646483	0,512309
11	Cont. LPS (EEP)	0,019566	0,000156	0,010077	0,000733	0,000495	0,011538	0,011250	0,011027	0,995536	0,646483		0,842671
12	Cont. LPS+IFN-γ (EEP)	0,012431	0,000094	0,006291	0,000442	0,000298	0,007226	0,007041	0,006899	0,847045	0,512309	0,842671	

Table S5. The effect of etanolic extract of Brazilian green propolis with LPS and/or IFN-α on the production of PDGF-BB in compared to control in CCF-STTG1 cells (n=3) in normoxia. Fisher's LSD test was used to evaluate statistical significance. Results marked in red are statistically significant in Fisher's LSD test. Multivariate Tests of Significance ($F = 6.009$, $p < 0.05$).

	LSD test; variable PDGF-BB<br>Probabilities for Post Hoc Tests<br>Error: Between MS = 20,287, df = 24,000												
Cell No.	Sample	{1}	{2}	{3}	{4}	{5}	{6}	{7}	{8}	{9}	{10}	{11}	{12}
1	EEP Brasil 25μg/ml		0,560778	0,999346	0,007877	0,662636	0,771922	0,471850	0,561914	0,252255	0,771922	0,999339	0,314800
2	EEP Brasil 25μg/ml+ IFN-γ	0,560778		0,560232	0,001893	0,312533	0,385971	0,198992	0,250323	0,090628	0,385971	0,561331	0,119038
3	EEP Brasil 25μg/ml+ LPS	0,999346	0,560232		0,007892	0,663227	0,772547	0,472347	0,562461	0,252581	0,772547	0,998685	0,315183
4	EEP Brasil 25μg/ml+ LPS+IFN-γ	0,007877	0,001893	0,007892		0,021604	0,015495	0,040292	0,029742	0,097209	0,015495	0,007862	0,073387
5	EEP Brasil 50μg/ml	0,662636	0,312533	0,663227	0,021604		0,883129	0,774855	0,884802	0,471618	0,883129	0,662038	0,564032
6	EEP Brasil 50μg/ml+ IFN-γ	0,771922	0,385971	0,772547	0,015495	0,883129		0,665409	0,770522	0,387600	1,000000	0,771289	0,470335
7	EEP Brasil 50μg/ml+ LPS	0,471850	0,198992	0,472347	0,040292	0,774855	0,665409		0,887608	0,662360	0,665409	0,471348	0,770012
8	EEP Brasil 50μg/ml+ LPS+IFN-γ	0,561914	0,250323	0,562461	0,029742	0,884802	0,770522	0,887608		0,564032	0,770522	0,561361	0,664925
9	Cont. (EEP)	0,252255	0,090628	0,252581	0,097209	0,471618	0,387600	0,662360	0,564032		0,387600	0,251926	0,884802
10	Cont. IFN-γ (EEP)	0,771922	0,385971	0,772547	0,015495	0,883129	1,000000	0,665409	0,770522	0,387600		0,771289	0,470335
11	Cont. LPS (EEP)	0,999339	0,561331	0,998685	0,007862	0,662038	0,771289	0,471348	0,561361	0,251926	0,771289		0,314414
12	Cont. LPS+IFN-γ (EEP)	0,314800	0,119038	0,315183	0,073387	0,564032	0,470335	0,770012	0,664925	0,884802	0,470335	0,314414	

Table S6. The effect of etanolic extract of Brazilian green propolis with LPS and/or IFN-α on the production of IL-6 in compared to control in CCF-STTG1 cells (n=3) in hypoxia. Fisher's LSD test was used to evaluate statistical significance. Results marked in red are statistically significant in Fisher's LSD test. Multivariate Tests of Significance ($F = 4.363$, $p < 0.05$).

	LSD test; variable IL-6<br>Probabilities for Post Hoc Tests<br>Error: Between MS = 9341,6, df = 24,000												
Cell No.	Sample	{1}	{2}	{3}	{4}	{5}	{6}	{7}	{8}	{9}	{10}	{11}	{12}
1	EEP Brasil 25µg/ml hypoxia		0,188606	0,156752	0,000000	0,232389	0,632155	0,884256	0,000001	0,000017	0,000000	0,000000	0,000000
2	EEP Brasil 25µg/ml+ IFN-γ hypoxia	0,188606		0,914368	0,000002	0,016489	0,393829	0,239552	0,000021	0,000524	0,000000	0,000000	0,000000
3	EEP Brasil 25µg/ml+ LPS hypoxia	0,156752	0,914368		0,000003	0,012883	0,338328	0,201028	0,000027	0,000689	0,000000	0,000000	0,000000
4	EEP Brasil 25µg/ml+ LPS+IFN-γ hypoxia	0,000000	0,000002	0,000003		0,000000	0,000000	0,000000	0,389674	0,042217	0,001767	0,259188	0,000000
5	EEP Brasil 50µg/ml hypoxia	0,232389	0,016489	0,012883	0,000000		0,100150	0,182650	0,000000	0,000001	0,000000	0,000000	0,000000
6	EEP Brasil 50µg/ml+ IFN-γ hypoxia	0,632155	0,393829	0,338328	0,000000	0,100150		0,738488	0,000002	0,000058	0,000000	0,000000	0,000000
7	EEP Brasil 50µg/ml+ LPS hypoxia	0,884256	0,239552	0,201028	0,000000	0,182650	0,738488		0,000001	0,000025	0,000000	0,000000	0,000000
8	EEP Brasil 50µg/ml+ LPS+IFN-γ hypoxia	0,000001	0,000021	0,000027	0,389674	0,000000	0,000002	0,000001		0,216375	0,000194	0,053391	0,000000
9	Cont. hypoxia (EEP)	0,000017	0,000524	0,000689	0,042217	0,000001	0,000058	0,000025	0,216375		0,000008	0,003001	0,000000
10	Cont. IFN-γ hypoxia (EEP)	0,000000	0,000000	0,000000	0,001767	0,000000	0,000000	0,000000	0,000194	0,000008		0,026678	0,000003
11	Cont. LPS hypoxia (EEP)	0,000000	0,000000	0,000000	0,259188	0,000000	0,000000	0,000000	0,053391	0,003001	0,026678		0,000000
12	Cont. LPS+IFN-γ hypoxia (EEP)	0,000000	0,000000	0,000000	0,000000	0,000000	0,000000	0,000000	0,000000	0,000000	0,000003	0,000000	

Table S7. The effect of etanolic extract of Brazilian green propolis with LPS and/or IFN-α on the production of IFN-γ in compared to control in CCF-STTG1 cells (n=3) in hypoxia. Fisher's LSD test was used to evaluate statistical significance. Results marked in red are statistically significant in Fisher's LSD test. Multivariate Tests of Significance ($F = 4.363$, $p < 0.05$).

	LSD test; variable IFN-γ<br>Probabilities for Post Hoc Tests<br>Error: Between MS = ,99318, df = 24,000												
Cell No.	Sample	{1}	{2}	{3}	{4}	{5}	{6}	{7}	{8}	{9}	{10}	{11}	{12}
1	EEP Brasil 25µg/ml hypoxia		0,000121	0,009996	0,000005	0,874513	0,000653	0,007787	0,000022	0,203870	0,000061	0,004129	0,000066
2	EEP Brasil 25µg/ml+ IFN-γ hypoxia	0,000121		0,087334	0,213839	0,000081	0,512631	0,106796	0,512174	0,003214	0,792168	0,171509	0,812697
3	EEP Brasil 25µg/ml+ LPS hypoxia	0,009996	0,087334		0,005387	0,006876	0,274708	0,915813	0,022066	0,149005	0,051559	0,712482	0,054459
4	EEP Brasil 25µg/ml+ LPS+IFN-γ hypoxia	0,000005	0,213839	0,005387		0,000003	0,064019	0,006944	0,546554	0,000130	0,322340	0,012901	0,309902
5	EEP Brasil 50µg/ml hypoxia	0,874513	0,000081	0,006876	0,000003		0,000436	0,005333	0,000015	0,155680	0,000041	0,002801	0,000044
6	EEP Brasil 50µg/ml+ IFN-γ hypoxia	0,000653	0,512631	0,274708	0,064019	0,000436		0,322104	0,196033	0,015396	0,361090	0,463564	0,374882
7	EEP Brasil 50µg/ml+ LPS hypoxia	0,007787	0,106796	0,915813	0,006944	0,005333	0,322104		0,027873	0,123179	0,063955	0,792442	0,067460
8	EEP Brasil 50µg/ml+ LPS+IFN-γ hypoxia	0,000022	0,512174	0,022066	0,546554	0,000015	0,196033	0,027873		0,000615	0,693499	0,048888	0,674067
9	Cont. hypoxia (EEP)	0,203870	0,003214	0,149005	0,000130	0,155680	0,015396	0,123179	0,000615		0,001669	0,074621	0,001784
10	Cont. IFN-γ hypoxia (EEP)	0,000061	0,792168	0,051559	0,322340	0,000041	0,361090	0,063955	0,693499	0,001669		0,106725	0,978778
11	Cont. LPS hypoxia (EEP)	0,004129	0,171509	0,712482	0,012901	0,002801	0,463564	0,792442	0,048888	0,074621	0,106725		0,112159
12	Cont. LPS+IFN-γ hypoxia (EEP)	0,000066	0,812697	0,054459	0,309902	0,000044	0,374882	0,067460	0,674067	0,001784	0,978778	0,112159	

Table S8. The effect of etanolic extract of Brazilian green propolis with LPS and/or IFN-α on the production of IL-5 in compared to control in CCF-STTG1 cells (n=3) in hypoxia. Fisher's LSD test was used to evaluate statistical significance. Results marked in red are statistically significant in Fisher's LSD test. Multivariate Tests of Significance ($F = 4.363$, $p < 0.05$).

	LSD test; variable IL-5<br>Probabilities for Post Hoc Tests<br>Error: Between MS = 793,52, df = 24,000												
Cell No.	Sample	{1}	{2}	{3}	{4}	{5}	{6}	{7}	{8}	{9}	{10}	{11}	{12}
1	EEP Brasil 25µg/ml hypoxia		0,163634	0,240644	0,000653	0,636419	0,067704	0,081075	0,005390	0,224942	0,001192	0,016453	0,000002
2	EEP Brasil 25µg/ml+ IFN-γ hypoxia	0,163634		0,817120	0,020634	0,347524	0,638114	0,704234	0,117832	0,849824	0,034759	0,264564	0,000059
3	EEP Brasil 25µg/ml+ LPS hypoxia	0,240644	0,817120		0,012170	0,475836	0,484435	0,542411	0,075778	0,966525	0,020921	0,181470	0,000033
4	EEP Brasil 25µg/ml+ LPS+IFN-γ hypoxia	0,000653	0,020634	0,012170		0,002156	0,056743	0,047007	0,400502	0,013411	0,812350	0,194173	0,025546
5	EEP Brasil 50µg/ml hypoxia	0,636419	0,347524	0,475836	0,002156		0,164316	0,192042	0,016417	0,450686	0,003876	0,046367	0,000005
6	EEP Brasil 50µg/ml+ IFN-γ hypoxia	0,067704	0,638114	0,484435	0,056743	0,164316		0,927300	0,263179	0,510648	0,090856	0,511759	0,000200
7	EEP Brasil 50µg/ml+ LPS hypoxia	0,081075	0,704234	0,542411	0,047007	0,192042	0,927300		0,227692	0,570264	0,076084	0,455707	0,000158
8	EEP Brasil 50µg/ml+ LPS+IFN-γ hypoxia	0,005390	0,117832	0,075778	0,400502	0,016417	0,263179	0,227692		0,082270	0,543755	0,635724	0,003511
9	Cont. hypoxia (EEP)	0,224942	0,849824	0,966525	0,013411	0,450686	0,510648	0,570264	0,082270		0,022975	0,194796	0,000037
10	Cont. IFN-γ hypoxia (EEP)	0,001192	0,034759	0,020921	0,812350	0,003876	0,090856	0,076084	0,543755	0,022975		0,284088	0,014972
11	Cont. LPS hypoxia (EEP)	0,016453	0,264564	0,181470	0,194173	0,046367	0,511759	0,455707	0,635724	0,194796	0,284088		0,001074
12	Cont. LPS+IFN-γ hypoxia (EEP)	0,000002	0,000059	0,000033	0,025546	0,000005	0,000200	0,000158	0,003511	0,000037	0,014972	0,001074	

Table S9. The effect of etanolic extract of Brazilian green propolis with LPS and/or IFN-α on the production of VEGF in compared to control in CCF-STTG1 cells (n=3) in hypoxia. Fisher's LSD test was used to evaluate statistical significance. Results marked in red are statistically significant in Fisher's LSD test. Multivariate Tests of Significance ($F = 4.363$, $p < 0.05$).

Cell No.	LSD test; variable VEGF<br>Probabilities for Post Hoc Tests<br>Error: Between MS = 711,45, df = 24,000												
	Sample	{1}	{2}	{3}	{4}	{5}	{6}	{7}	{8}	{9}	{10}	{11}	{12}
1	EEP Brasil 25µg/ml hypoxia		0,315675	0,688947	0,504744	0,960430	0,946598	0,647988	0,012337	0,000073	0,000269	0,004191	0,000006
2	EEP Brasil 25µg/ml+ IFN-γ hypoxia	0,315675		0,541333	0,731182	0,339447	0,285465	0,579031	0,105701	0,000981	0,003486	0,042808	0,000075
3	EEP Brasil 25µg/ml+ LPS hypoxia	0,688947	0,541333		0,787906	0,725670	0,640595	0,954861	0,030397	0,000205	0,000751	0,010926	0,000016
4	EEP Brasil 25µg/ml+ LPS+IFN-γ hypoxia	0,504744	0,731182	0,787906		0,536525	0,463564	0,831693	0,053718	0,000408	0,001483	0,020246	0,000031
5	EEP Brasil 50µg/ml hypoxia	0,960430	0,339447	0,725670	0,536525		0,907192	0,683834	0,013835	0,000083	0,000306	0,004729	0,000006
6	EEP Brasil 50µg/ml+ IFN-γ hypoxia	0,946598	0,285465	0,640595	0,463564	0,907192		0,600952	0,010555	0,000062	0,000227	0,003558	0,000005
7	EEP Brasil 50µg/ml+ LPS hypoxia	0,647988	0,579031	0,954861	0,831693	0,683834	0,600952		0,034353	0,000237	0,000867	0,012463	0,000018
8	EEP Brasil 50µg/ml+ LPS+IFN-γ hypoxia	0,012337	0,105701	0,030397	0,053718	0,013835	0,010555	0,034353		0,049183	0,132139	0,651135	0,005050
9	Cont. hypoxia (EEP)	0,000073	0,000981	0,000205	0,000408	0,000083	0,000062	0,000237	0,049183		0,612536	0,119596	0,320512
10	Cont. IFN-γ hypoxia (EEP)	0,000269	0,003486	0,000751	0,001483	0,000306	0,000227	0,000867	0,132139	0,612536		0,281874	0,139698
11	Cont. LPS hypoxia (EEP)	0,004191	0,042808	0,010926	0,020246	0,004729	0,003558	0,012463	0,651135	0,119596	0,281874		0,014725
12	Cont. LPS+IFN-γ hypoxia (EEP)	0,000006	0,000075	0,000016	0,000031	0,000006	0,000005	0,000018	0,005050	0,320512	0,139698	0,014725	

Table S10. The effect of etanolic extract of Brazilian green propolis with LPS and/or IFN-α on the production of PDGF-BB in compared to control in CCF-STTG1 cells (n=3) in hypoxia. Fisher's LSD test was used to evaluate statistical significance. Results marked in red are statistically significant in Fisher's LSD test. Multivariate Tests of Significance ($F = 4.363$, $p < 0.05$).

	LSD test; variable PDGF-BB<br>Probabilities for Post Hoc Tests<br>Error: Between MS = 29,980, df = 24,000												
Cell No.	Sample	{1}	{2}	{3}	{4}	{5}	{6}	{7}	{8}	{9}	{10}	{11}	{12}
1	EEP Brasil 25µg/ml hypoxia		0,479397	0,478724	0,013726	0,637259	0,629375	0,065838	0,065749	0,904034	0,040070	0,198613	0,003283
2	EEP Brasil 25µg/ml+ IFN-γ hypoxia	0,479397		0,999122	0,064112	0,811689	0,820335	0,238445	0,238189	0,556339	0,159386	0,551792	0,017729
3	EEP Brasil 25µg/ml+ LPS hypoxia	0,478724	0,999122		0,064254	0,810838	0,819481	0,238865	0,238608	0,555608	0,159692	0,552519	0,017773
4	EEP Brasil 25µg/ml+ LPS+IFN-γ hypoxia	0,013726	0,064112	0,064254		0,039155	0,040092	0,471347	0,471755	0,018077	0,629560	0,193674	0,550654
5	EEP Brasil 50µg/ml hypoxia	0,637259	0,811689	0,810838	0,039155		0,991102	0,160038	0,159850	0,725137	0,103375	0,406754	0,010233
6	EEP Brasil 50µg/ml+ IFN-γ hypoxia	0,629375	0,820335	0,819481	0,040092	0,991102		0,163178	0,162987	0,716823	0,105566	0,412954	0,010503
7	EEP Brasil 50µg/ml+ LPS hypoxia	0,065838	0,238445	0,238865	0,471347	0,160038	0,163178		0,999462	0,083538	0,809878	0,550587	0,193706
8	EEP Brasil 50µg/ml+ LPS+IFN-γ hypoxia	0,065749	0,238189	0,238608	0,471755	0,159850	0,162987	0,999462		0,083429	0,810400	0,550142	0,193926
9	Cont. hypoxia (EEP)	0,904034	0,556339	0,555608	0,018077	0,725137	0,716823	0,083538	0,083429		0,051561	0,241774	0,004410
10	Cont. IFN-γ hypoxia (EEP)	0,040070	0,159386	0,159692	0,629560	0,103375	0,105566	0,809878	0,810400	0,051561		0,404454	0,284852
11	Cont. LPS hypoxia (EEP)	0,198613	0,551792	0,552519	0,193674	0,406754	0,412954	0,550587	0,550142	0,241774	0,404454		0,063890
12	Cont. LPS+IFN-γ hypoxia (EEP)	0,003283	0,017729	0,017773	0,550654	0,010233	0,010503	0,193706	0,193926	0,004410	0,284852	0,063890	

Table S11. The effect of artepilin C with LPS and/or IFN-α on the production of IL-6 in compared to control in CCF-STTG1 cells (n=3) in normoxia. Fisher's LSD test was used to evaluate statistical significance. Results marked in red are statistically significant in Fisher's LSD test. Multivariate Tests of Significance ($F = 4.495$, $p < 0.05$).

	LSD test; variable IL-6<br>Probabilities for Post Hoc Tests<br>Error: Between MS = 17592,, df = 24,000												
Cell No.	Sample	{1}	{2}	{3}	{4}	{5}	{6}	{7}	{8}	{9}	{10}	{11}	{12}
1	Art. C 25µg/mL		0,060484	0,073666	0,000000	0,301269	0,000157	0,000181	0,000000	0,704449	0,000065	0,000854	0,000001
2	Art. C 25µg/m+ IFN-γ	0,060484		0,921537	0,000000	0,005827	0,019343	0,021884	0,000000	0,125804	0,008820	0,078419	0,000175
3	Art. C 25µg/m+ LPS	0,073666	0,921537		0,000000	0,007377	0,015468	0,017534	0,000000	0,150148	0,006980	0,064488	0,000136
4	Art. C 25µg/m+ LPS+IFN-γ	0,000000	0,000000	0,000000		0,000000	0,000010	0,000009	0,593092	0,000000	0,000024	0,000002	0,001318
5	Art. C 50µg/mL	0,301269	0,005827	0,007377	0,000000		0,000011	0,000012	0,000000	0,162686	0,000005	0,000059	0,000000
6	Art. C 50µg/mL+ IFN-γ	0,000157	0,019343	0,015468	0,000010	0,000011		0,956093	0,000039	0,000416	0,734092	0,509992	0,065931
7	Art. C 50µg/mL+ LPS	0,000181	0,021884	0,017534	0,000009	0,000012	0,956093		0,000034	0,000479	0,693204	0,545521	0,058997
8	Art. C 50µg/mL+ LPS+IFN-γ	0,000000	0,000000	0,000000	0,593092	0,000000	0,000039	0,000034		0,000000	0,000095	0,000007	0,004966
9	Cont. (ART.)	0,704449	0,125804	0,150148	0,000000	0,162686	0,000416	0,000479	0,000000		0,000174	0,002220	0,000003
10	Cont. IFN-γ (ART.)	0,000065	0,008820	0,006980	0,000024	0,000005	0,734092	0,693204	0,000095	0,000174		0,321406	0,126491
11	Cont. LPS (ART.)	0,000854	0,078419	0,064488	0,000002	0,000059	0,509992	0,545521	0,000007	0,002220	0,321406		0,015862
12	Cont. LPS+IFN-γ (ART.)	0,000001	0,000175	0,000136	0,001318	0,000000	0,065931	0,058997	0,004966	0,000003	0,126491	0,015862	

Table S12. The effect of artepilin C with LPS and/or IFN-α on the production of IFN-γ in compared to control in CCF-STTG1 cells (n=3) in normoxia. Fisher's LSD test was used to evaluate statistical significance. Results marked in red are statistically significant in Fisher's LSD test. Multivariate Tests of Significance (F = 4.495, p < 0.05).

Cell No.	LSD test; variable IFN-γ<br>Probabilities for Post Hoc Tests<br>Error: Between MS = ,95326, df = 24,000												
	Sample	{1}	{2}	{3}	{4}	{5}	{6}	{7}	{8}	{9}	{10}	{11}	{12}
1	Art. C 25μg/mL		0,036407	0,744982	0,036405	0,588177	0,587628	0,110813	0,087705	0,357906	0,283637	0,015793	0,000183
2	Art. C 25μg/mL+ IFN-γ	0,036407		0,017764	0,999977	0,010766	0,108601	0,000728	0,666729	0,004296	0,274011	0,706350	0,037684
3	Art. C 25μg/mL+ LPS	0,744982	0,017764		0,017763	0,827873	0,388276	0,197124	0,045541	0,548681	0,166810	0,007384	0,000079
4	Art. C 25μg/mL+ LPS+IFN-γ	0,036405	0,999977	0,017763		0,010765	0,108595	0,000728	0,666708	0,004296	0,273999	0,706371	0,037686
5	Art. C 50μg/mL	0,588177	0,010766	0,827873	0,010765		0,282877	0,279354	0,028599	0,701058	0,112882	0,004372	0,000045
6	Art. C 50μg/mL+ IFN-γ	0,587628	0,108601	0,388276	0,108595	0,282877		0,037257	0,230413	0,150026	0,589378	0,051669	0,000738
7	Art. C 50μg/mL+ LPS	0,110813	0,000728	0,197124	0,000728	0,279354	0,037257		0,002158	0,479522	0,011087	0,000277	0,000003
8	Art. C 50μg/mL+ LPS+IFN-γ	0,087705	0,666729	0,045541	0,666708	0,028599	0,230413	0,002158		0,012012	0,500849	0,421804	0,014477
9	Cont. (ART.)	0,357906	0,004296	0,548681	0,004296	0,701058	0,150026	0,479522	0,012012		0,053136	0,001690	0,000017
10	Cont. IFN-γ (ART.)	0,283637	0,274011	0,166810	0,273999	0,112882	0,589378	0,011087	0,500849	0,053136		0,146458	0,002873
11	Cont. LPS (ART.)	0,015793	0,706350	0,007384	0,706371	0,004372	0,051669	0,000277	0,421804	0,001690	0,146458		0,081470
12	Cont. LPS+IFN-γ (ART.)	0,000183	0,037684	0,000079	0,037686	0,000045	0,000738	0,000003	0,014477	0,000017	0,002873	0,081470	

Table S13. The effect of artepilin C with LPS and/or IFN-α on the production of IL-5 in compared to control in CCF-STTG1 cells (n=3) in normoxia. Fisher's LSD test was used to evaluate statistical significance. Results marked in red are statistically significant in Fisher's LSD test. Multivariate Tests of Significance (F = 4.495, $p < 0.05$).

	LSD test; variable IL-5<br>Probabilities for Post Hoc Tests<br>Error: Between MS = 514,64, df = 24,000												
Cell No.	Sample	{1}	{2}	{3}	{4}	{5}	{6}	{7}	{8}	{9}	{10}	{11}	{12}
1	Art. C 25µg/mL		0,029584	0,780267	0,000233	0,553364	0,036421	0,260429	0,000284	0,331955	0,001673	0,042948	0,001457
2	Art. C 25µg/mL+ IFN-γ	0,029584		0,015864	0,055928	0,007596	0,923265	0,257140	0,065523	0,198240	0,232271	0,861865	0,212343
3	Art. C 25µg/mL+ LPS	0,780267	0,015864		0,000114	0,752453	0,019734	0,164272	0,000139	0,215447	0,000827	0,023477	0,000719
4	Art. C 25µg/mL+ LPS+IFN-γ	0,000233	0,055928	0,000114		0,000050	0,045829	0,004133	0,937692	0,002785	0,441081	0,038906	0,473891
5	Art. C 50µg/mL	0,553364	0,007596	0,752453	0,000050		0,009539	0,092235	0,000062	0,124614	0,000370	0,011443	0,000321
6	Art. C 50µg/mL+ IFN-γ	0,036421	0,923265	0,019734	0,045829	0,009539		0,298157	0,053886	0,232147	0,198349	0,938050	0,180733
7	Art. C 50µg/mL+ LPS	0,260429	0,257140	0,164272	0,004133	0,092235	0,298157		0,004998	0,872357	0,025254	0,334489	0,022346
8	Art. C 50µg/mL+ LPS+IFN-γ	0,000284	0,065523	0,000139	0,937692	0,000062	0,053886	0,004998		0,003377	0,487987	0,045872	0,522745
9	Cont. (ART.)	0,331955	0,198240	0,215447	0,002785	0,124614	0,232147	0,872357	0,003377		0,017627	0,262561	0,015547
10	Cont. IFN-γ (ART.)	0,001673	0,232271	0,000827	0,441081	0,000370	0,198349	0,025254	0,487987	0,017627		0,173895	0,956006
11	Cont. LPS (ART.)	0,042948	0,861865	0,023477	0,038906	0,011443	0,938050	0,334489	0,045872	0,262561	0,173895		0,158038
12	Cont. LPS+IFN-γ (ART.)	0,001457	0,212343	0,000719	0,473891	0,000321	0,180733	0,022346	0,522745	0,015547	0,956006	0,158038	

Table S14. The effect of artepilin C with LPS and/or IFN-α on the production of VEGF in compared to control in CCF-STTG1 cells (n=3) in normoxia. Fisher's LSD test was used to evaluate statistical significance. Results marked in red are statistically significant in Fisher's LSD test. Multivariate Tests of Significance ($F = 4.495$, $p < 0.05$).

	LSD test; variable VEGF<br>Probabilities for Post Hoc Tests<br>Error: Between MS = 897,60, df = 24,000												
Cell No.	Sample	{1}	{2}	{3}	{4}	{5}	{6}	{7}	{8}	{9}	{10}	{11}	{12}
1	Art. C 25µg/mL		0,156264	0,634440	0,039341	0,706765	0,048778	0,137322	0,027022	0,027262	0,002099	0,120061	0,001614
2	Art. C 25µg/mL+ IFN-γ	0,156264		0,335879	0,481014	0,077509	0,546092	0,941971	0,381455	0,383599	0,058859	0,883398	0,047408
3	Art. C 25µg/mL+ LPS	0,634440	0,335879		0,102467	0,397041	0,123945	0,301664	0,073207	0,073792	0,006734	0,269533	0,005229
4	Art. C 25µg/mL+ LPS+IFN-γ	0,039341	0,481014	0,102467		0,017179	0,918422	0,526796	0,861930	0,865095	0,217055	0,575591	0,182142
5	Art. C 50µg/mL	0,706765	0,077509	0,397041	0,017179		0,021637	0,067095	0,011514	0,011623	0,000813	0,057796	0,000622
6	Art. C 50µg/mL+ IFN-γ	0,048778	0,546092	0,123945	0,918422	0,021637		0,595022	0,782408	0,785497	0,182987	0,646775	0,152546
7	Art. C 50µg/mL+ LPS	0,137322	0,941971	0,301664	0,526796	0,067095	0,595022		0,421377	0,423661	0,068156	0,941094	0,055099
8	Art. C 50µg/mL+ LPS+IFN-γ	0,027022	0,381455	0,073207	0,861930	0,011514	0,782408	0,421377		0,996784	0,285700	0,464470	0,242540
9	Cont. (ART.)	0,027262	0,383599	0,073792	0,865095	0,011623	0,785497	0,423661	0,996784		0,283950	0,466893	0,240988
10	Cont. IFN-γ (ART.)	0,002099	0,058859	0,006734	0,217055	0,000813	0,182987	0,068156	0,285700	0,283950		0,078882	0,916262
11	Cont. LPS (ART.)	0,120061	0,883398	0,269533	0,575591	0,057796	0,646775	0,941094	0,464470	0,466893	0,078882		0,064018
12	Cont. LPS+IFN-γ (ART.)	0,001614	0,047408	0,005229	0,182142	0,000622	0,152546	0,055099	0,242540	0,240988	0,916262	0,064018	

Table S15. The effect of artepilin C with LPS and/or IFN-α on the production of PDGF-BB in compared to control in CCF-STTG1 cells (n=3) in normoxia. Fisher's LSD test was used to evaluate statistical significance. Results marked in red are statistically significant in Fisher's LSD test. Multivariate Tests of Significance (F = 4.495, p < 0.05).

	LSD test; variable PDGF-BB<br>Probabilities for Post Hoc Tests<br>Error: Between MS = 23,836, df = 24,000												
Cell No.	Sample	{1}	{2}	{3}	{4}	{5}	{6}	{7}	{8}	{9}	{10}	{11}	{12}
1	Art. C 25μg/mL		0,768630	0,903894	0,901034	0,604242	0,346254	0,515624	0,502321	0,602037	0,765508	0,605839	0,356318
2	Art. C 25μg/mL+ IFN-γ	0,768630		0,678549	0,675920	0,418751	0,513499	0,347920	0,704658	0,416954	0,996729	0,420052	0,227673
3	Art. C 25μg/mL+ LPS	0,903894	0,678549		0,997117	0,690368	0,289664	0,595642	0,429778	0,688029	0,675567	0,692060	0,421124
4	Art. C 25μg/mL+ LPS+IFN-γ	0,901034	0,675920	0,997117		0,693020	0,288078	0,598127	0,427712	0,690677	0,672942	0,694715	0,423170
5	Art. C 50μg/mL	0,604242	0,418751	0,690368	0,693020		0,150301	0,894024	0,239443	0,997454	0,416444	0,998159	0,681613
6	Art. C 50μg/mL+ IFN-γ	0,346254	0,513499	0,289664	0,288078	0,150301		0,118165	0,782145	0,149456	0,516109	0,150915	0,069337
7	Art. C 50μg/mL+ LPS	0,515624	0,347920	0,595642	0,598127	0,894024	0,118165		0,192483	0,896547	0,345876	0,892201	0,781373
8	Art. C 50μg/mL+ LPS+IFN-γ	0,502321	0,704658	0,429778	0,427712	0,239443	0,782145	0,192483		0,238226	0,707691	0,240327	0,117946
9	Cont. (ART.)	0,602037	0,416954	0,688029	0,690677	0,997454	0,149456	0,896547	0,238226		0,414654	0,995613	0,683943
10	Cont. IFN-γ (ART.)	0,765508	0,996729	0,675567	0,672942	0,416444	0,516109	0,345876	0,707691	0,414654		0,417741	0,226168
11	Cont. LPS (ART.)	0,605839	0,420052	0,692060	0,694715	0,998159	0,150915	0,892201	0,240327	0,995613	0,417741		0,679931
12	Cont. LPS+IFN-γ (ART.)	0,356318	0,227673	0,421124	0,423170	0,681613	0,069337	0,781373	0,117946	0,683943	0,226168	0,679931	

Table S16. The effect of artepilin C with LPS and/or IFN-α on the production of IL-6 in compared to control in CCF-STTG1 cells (n=3) in hypoxia. Fisher's LSD test was used to evaluate statistical significance. Results marked in red are statistically significant in Fisher's LSD test. Multivariate Tests of Significance (F = 2.5011, p < 0.05).

	LSD test; variable IL-6<br>Probabilities for Post Hoc Tests<br>Error: Between MS = 36771,, df = 24,000												
Cell No.	Sample	{1}	{2}	{3}	{4}	{5}	{6}	{7}	{8}	{9}	{10}	{11}	{12}
1	Art. C 25µg/mL hypoxia		0,005869	0,170761	0,000000	0,963234	0,039729	0,075122	0,000002	0,704264	0,006062	0,134392	0,000009
2	Art. C 25µg/mL+ IFN-γ hypoxia	0,005869		0,120167	0,000219	0,006555	0,404438	0,256243	0,003093	0,014364	0,989231	0,153435	0,015916
3	Art. C 25µg/mL+ LPS hypoxia	0,170761	0,120167		0,000004	0,184757	0,453070	0,657923	0,000054	0,314221	0,123180	0,891989	0,000313
4	Art. C 25µg/mL LPS+IFN-γ hypoxia	0,000000	0,000219	0,000004		0,000000	0,000025	0,000012	0,301356	0,000000	0,000212	0,000005	0,092666
5	Art. C 50µg/mL hypoxia	0,963234	0,006555	0,184757	0,000000		0,043786	0,082224	0,000002	0,738635	0,006771	0,145951	0,000010
6	Art. C 50µg/mL+ IFN-γ hypoxia	0,039729	0,404438	0,453070	0,000025	0,043786		0,755966	0,000372	0,085971	0,411923	0,537565	0,002122
7	Art. C 50µg/mL+ LPS hypoxia	0,075122	0,256243	0,657923	0,000012	0,082224	0,755966		0,000167	0,152864	0,261712	0,758401	0,000971
8	Art. C 50µg/mL+ LPS+IFN-γ hypoxia	0,000002	0,003093	0,000054	0,301356	0,000002	0,000372	0,000167		0,000004	0,002992	0,000076	0,493718
9	Cont. hypoxia (ART.)	0,704264	0,014364	0,314221	0,000000	0,738635	0,085971	0,152864	0,000004		0,014816	0,255393	0,000023
10	Cont. IFN-γ hypoxia (ART.)	0,006062	0,989231	0,123180	0,000212	0,006771	0,411923	0,261712	0,002992	0,014816		0,157114	0,015433
11	Cont. LPS hypoxia (ART.)	0,134392	0,153435	0,891989	0,000005	0,145951	0,537565	0,758401	0,000076	0,255393	0,157114		0,000443
12	Cont. LPS+IFN-γ hypoxia (ART.)	0,000009	0,015916	0,000313	0,092666	0,000010	0,002122	0,000971	0,493718	0,000023	0,015433	0,000443	

Table S17. The effect of artepilin C with LPS and/or IFN-α on the production of IL-6 in compared to control in CCF-STTG1 cells (n=3) in hypoxia. Fisher's LSD test was used to evaluate statistical significance. Results marked in red are statistically significant in Fisher's LSD test. Multivariate Tests of Significance (F = 2.5011, p < 0.05).

	LSD test; variable IFN-γ<br>Probabilities for Post Hoc Tests<br>Error: Between MS = 11,838, df = 24,000												
Cell No.	Sample	{1}	{2}	{3}	{4}	{5}	{6}	{7}	{8}	{9}	{10}	{11}	{12}
1	Art. C 25µM hypoxia		0,436841	0,907663	0,510644	0,769634	0,623773	0,757512	0,555579	0,944902	0,445424	0,724033	0,001397
2	Art. C 25µg/mL+ IFN-γ hypoxia	0,436841		0,507050	0,903165	0,287865	0,771416	0,280949	0,848623	0,397987	0,988233	0,668525	0,009461
3	Art. C 25µg/mL+ LPS hypoxia	0,907663	0,507050		0,587021	0,682980	0,707512	0,671380	0,635172	0,853190	0,516409	0,812349	0,001869
4	Art. C 25µg/mL+ LPS+IFN-γ hypoxia	0,510644	0,903165	0,587021		0,344674	0,865758	0,336824	0,944780	0,467897	0,914851	0,758831	0,007091
5	Art. C 50µg/mL hypoxia	0,769634	0,287865	0,682980	0,344674		0,435491	0,987281	0,380211	0,822845	0,294371	0,519690	0,000665
6	Art. C 50µg/mL+ IFN-γ hypoxia	0,623773	0,771416	0,707512	0,865758	0,435491		0,426345	0,920484	0,576147	0,782691	0,890087	0,004719
7	Art. C 50µg/mL+ LPS hypoxia	0,757512	0,280949	0,671380	0,336824	0,987281	0,426345		0,371826	0,810484	0,287343	0,509542	0,000639
8	Art. C 50µg/mL+ LPS+IFN-γ hypoxia	0,555579	0,848623	0,635172	0,944780	0,380211	0,920484	0,371826		0,510740	0,860181	0,811948	0,006007
9	Cont. hypoxia (ART.)	0,944902	0,397987	0,853190	0,467897	0,822845	0,576147	0,810484	0,510740		0,406087	0,673128	0,001174
10	Cont. IFN-γ hypoxia (ART.)	0,445424	0,988233	0,516409	0,914851	0,294371	0,782691	0,287343	0,860181	0,406087		0,679235	0,009138
11	Cont. LPS hypoxia (ART.)	0,724033	0,668525	0,812349	0,758831	0,519690	0,890087	0,509542	0,811948	0,673128	0,679235		0,003367
12	Cont. LPS+IFN-γ hypoxia (ART.)	0,001397	0,009461	0,001869	0,007091	0,000665	0,004719	0,000639	0,006007	0,001174	0,009138	0,003367	

Table S18. The effect of artepilin C with LPS and/or IFN-α on the production of IL-5 in compared to control in CCF-STTG1 cells (n=3) in hypoxia. Fisher's LSD test was used to evaluate statistical significance. Results marked in red are statistically significant in Fisher's LSD test. Multivariate Tests of Significance (F = 2.5011, p < 0.05).

Cell No.	LSD test; variable IL-5<br>Probabilities for Post Hoc Tests<br>Error: Between MS = 704,91, df = 24,000												
	Sample	{1}	{2}	{3}	{4}	{5}	{6}	{7}	{8}	{9}	{10}	{11}	{12}
1	Art. C 25µg/mL hypoxia		0,077937	0,097590	0,000078	0,487778	0,122781	0,133345	0,000392	0,412385	0,054176	0,152932	0,000554
2	Art. C 25µg/mL+ IFN-γ hypoxia	0,077937		0,907323	0,007637	0,017725	0,810856	0,775950	0,032084	0,323834	0,856301	0,717954	0,042841
3	Art. C 25µg/mL+ LPS hypoxia	0,097590	0,907323		0,005780	0,023020	0,902095	0,866298	0,024873	0,382480	0,766236	0,806374	0,033433
4	Art. C 25µg/mL+ LPS+IFN-γ hypoxia	0,000078	0,007637	0,005780		0,000013	0,004290	0,003840	0,530407	0,000646	0,011695	0,003180	0,446758
5	Art. C 50µg/mL hypoxia	0,487778	0,017725	0,023020	0,000013		0,030172	0,033287	0,000065	0,136905	0,011693	0,039235	0,000093
6	Art. C 50µg/mL+ IFN-γ hypoxia	0,122781	0,810856	0,902095	0,004290	0,030172		0,963806	0,018898	0,451532	0,674610	0,902734	0,025567
7	Art. C 50µg/mL+ LPS hypoxia	0,133345	0,775950	0,866298	0,003840	0,033287	0,963806		0,017053	0,478791	0,641984	0,938748	0,023124
8	Art. C 50µg/mL+ LPS+IFN-γ hypoxia	0,000392	0,032084	0,024873	0,530407	0,000065	0,018898	0,017053		0,003140	0,047137	0,014307	0,892228
9	Cont. hypoxia (ART.)	0,412385	0,323834	0,382480	0,000646	0,136905	0,451532	0,478791	0,003140		0,245549	0,527067	0,004376
10	Cont. IFN-γ hypoxia (ART.)	0,054176	0,856301	0,766236	0,011695	0,011693	0,674610	0,641984	0,047137	0,245549		0,588398	0,062244
11	Cont. LPS hypoxia (ART.)	0,152932	0,717954	0,806374	0,003180	0,039235	0,902734	0,938748	0,014307	0,527067	0,588398		0,019472
12	Cont. LPS+IFN-γ hypoxia (ART.)	0,000554	0,042841	0,033433	0,446758	0,000093	0,025567	0,023124	0,892228	0,004376	0,062244	0,019472	

Table S19. The effect of artepilin C with LPS and/or IFN-α on the production of VEGF in compared to control in CCF-STTG1 cells (n=3) in hypoxia. Fisher's LSD test was used to evaluate statistical significance. Results marked in red are statistically significant in Fisher's LSD test. Multivariate Tests of Significance ($F = 2.5011$, $p < 0.05$).

	LSD test; variable VEGF<br>Probabilities for Post Hoc Tests<br>Error: Between MS = 968,42, df = 24,000												
Cell No.	Sample	{1}	{2}	{3}	{4}	{5}	{6}	{7}	{8}	{9}	{10}	{11}	{12}
1	Art. C 25µg/mL hypoxia		0,046353	0,182149	0,018267	0,695804	0,857380	0,659318	0,018910	0,027387	0,003067	0,327176	0,000361
2	Art. C 25µg/mL+ IFN-γ hypoxia	0,046353		0,474409	0,669535	0,101108	0,031624	0,111082	0,680676	0,805971	0,244981	0,282037	0,051591
3	Art. C 25µg/mL+ LPS hypoxia	0,182149	0,474409		0,257924	0,337712	0,132888	0,362861	0,264172	0,339234	0,067008	0,711883	0,010514
4	Art. C 25µg/mL+ LPS+IFN-γ hypoxia	0,018267	0,669535	0,257924		0,042986	0,012098	0,047740	0,987769	0,855768	0,454808	0,138458	0,119058
5	Art. C 50µg/mL hypoxia	0,695804	0,101108	0,337712	0,042986		0,569061	0,960012	0,044393	0,062532	0,007919	0,551178	0,000980
6	Art. C 50µg/mL+ IFN-γ hypoxia	0,857380	0,031624	0,132888	0,012098	0,569061		0,535907	0,012536	0,018352	0,001964	0,248829	0,000228
7	Art. C 50µg/mL+ LPS hypoxia	0,659318	0,111082	0,362861	0,047740	0,960012	0,535907		0,049285	0,069146	0,008917	0,584810	0,001112
8	Art. C 50µg/mL+ LPS+IFN-γ hypoxia	0,018910	0,680676	0,264172	0,987769	0,044393	0,012536	0,049285		0,867804	0,445775	0,142309	0,115736
9	Cont. hypoxia (ART.)	0,027387	0,805971	0,339234	0,855768	0,062532	0,018352	0,069146	0,867804		0,354841	0,189978	0,084408
10	Cont. IFN-γ hypoxia (ART.)	0,003067	0,244981	0,067008	0,454808	0,007919	0,001964	0,008917	0,445775	0,354841		0,030957	0,400055
11	Cont. LPS hypoxia (ART.)	0,327176	0,282037	0,711883	0,138458	0,551178	0,248829	0,584810	0,142309	0,189978	0,030957		0,004344
12	Cont. LPS+IFN-γ hypoxia (ART.)	0,000361	0,051591	0,010514	0,119058	0,000980	0,000228	0,001112	0,115736	0,084408	0,400055	0,004344	

Table S20. The effect of artepilin C with LPS and/or IFN-α on the production of PDGF-BB in compared to control in CCF-STTG1 cells (n=3) in hypoxia. Fisher's LSD test was used to evaluate statistical significance. Results marked in red are statistically significant in Fisher's LSD test. Multivariate Tests of Significance ($F = 2.5011$, $p < 0.05$).

Cell No.	LSD test; variable PDGF-BB<br>Probabilities for Post Hoc Tests<br>Error: Between MS = 17,877, df = 24,000												
	Sample	{1}	{2}	{3}	{4}	{5}	{6}	{7}	{8}	{9}	{10}	{11}	{12}
1	Art. C 25µg/mL hypoxia		0,347352	0,530595	0,998530	0,759245	0,998530	0,429192	0,353033	0,339476	0,137876	0,528890	0,530336
2	Art. C 25µg/mL+ IFN-γ hypoxia	0,347352		0,123833	0,348272	0,522791	0,346433	0,090682	0,990963	0,065100	0,569702	0,752070	0,750378
3	Art. C 25µg/mL+ LPS hypoxia	0,530595	0,123833		0,529403	0,353426	0,531789	0,868100	0,126420	0,738082	0,040024	0,214414	0,215201
4	Art. C 25µg/mL+ LPS+IFN-γ hypoxia	0,998530	0,348272	0,529403		0,760644	0,997060	0,428138	0,353964	0,338572	0,138333	0,530081	0,531529
5	Art. C 50µg/mL hypoxia	0,759245	0,522791	0,353426	0,760644		0,757846	0,276244	0,530092	0,211194	0,232478	0,745019	0,746707
6	Art. C 50µg/mL+ IFN-γ hypoxia	0,998530	0,346433	0,531789	0,997060	0,757846		0,430247	0,352105	0,340382	0,137420	0,527700	0,529144
7	Art. C 50µg/mL+ LPS hypoxia	0,429192	0,090682	0,868100	0,428138	0,276244	0,430247		0,092674	0,866096	0,027985	0,161905	0,162536
8	Art. C 50µg/mL+ LPS+IFN-γ hypoxia	0,353033	0,990963	0,126420	0,353964	0,530092	0,352105	0,092674		0,066596	0,562118	0,760656	0,758958
9	Cont. hypoxia (ART.)	0,339476	0,065100	0,738082	0,338572	0,211194	0,340382	0,866096	0,066596		0,019240	0,119690	0,120184
10	Cont. IFN-γ hypoxia (ART.)	0,137876	0,569702	0,040024	0,138333	0,232478	0,137420	0,027985	0,562118	0,019240		0,379168	0,377986
11	Cont. LPS hypoxia (ART.)	0,528890	0,752070	0,214414	0,530081	0,745019	0,527700	0,161905	0,760656	0,119690	0,379168		0,998216
12	Cont. LPS+IFN-γ hypoxia (ART.)	0,530336	0,750378	0,215201	0,531529	0,746707	0,529144	0,162536	0,758958	0,120184	0,377986	0,998216	

Disclaimer/Publisher's Note: The statements, opinions and data contained in all publications are solely those of the individual author(s) and contributor(s) and not of MDPI and/or the editor(s). MDPI and/or the editor(s) disclaim responsibility for any injury to people or property resulting from any ideas, methods, instructions or products referred to in the content.
